# Elucidating the mechanisms by which disulfiram protects against obesity and metabolic syndrome

**DOI:** 10.1038/s41514-020-0046-6

**Published:** 2020-07-21

**Authors:** Michel Bernier, Dylan Harney, Yen Chin Koay, Antonio Diaz, Abhishek Singh, Devin Wahl, Tamara Pulpitel, Ahmed Ali, Vince Guiterrez, Sarah J. Mitchell, Eun-Young Kim, John Mach, Nathan L. Price, Miguel A. Aon, David G. LeCouteur, Victoria C. Cogger, Carlos Fernandez-Hernando, John O’Sullivan, Mark Larance, Ana Maria Cuervo, Rafael de Cabo

**Affiliations:** 1 Experimental Gerontology Section, Translational Gerontology Branch, National Institute on Aging, National Institutes of Health, Baltimore, MD 21224 USA; 2 Charles Perkins Centre, The University of Sydney, Sydney, NSW 2006 Australia; 3 Heart Research Institute, The University of Sydney, Sydney, NSW 2042 Australia; 4 Department of Developmental and Molecular Biology, Institute for Aging Studies, Albert Einstein College of Medicine, New York, NY 10461 USA; 5 Vascular Biology and Therapeutics Program, Integrative Cell Signaling and Neurobiology of Metabolism Program, Department of Comparative Medicine, Department of Pathology, Yale University School of Medicine, New Haven, CT 06510 USA; 6 Ageing and Alzheimer’s Institute, ANZAC Research Institute, Concord Clinical School/Sydney Medical School, Concord, NSW 2139 Australia; 7 Functional Genomics Research Center, KRIBB, Daejeon, 305-806 Republic of Korea; 8 Kolling Institute of Medical Research and Sydney Medical School, University of Sydney, Sydney, NSW 2064 Australia

**Keywords:** Metabolic syndrome, Obesity

## Abstract

There is an unmet need and urgency to find safe and effective anti-obesity interventions. Our recent study in mice fed on obesogenic diet found that treatment with the alcohol aversive drug disulfiram reduced feeding efficiency and led to a decrease in body weight and an increase in energy expenditure. The intervention with disulfiram improved glucose tolerance and insulin sensitivity, and mitigated metabolic dysfunctions in various organs through poorly defined mechanisms. Here, integrated analysis of transcriptomic and proteomic data from mouse and rat livers unveiled comparable signatures in response to disulfiram, revealing pathways associated with lipid and energy metabolism, redox, and detoxification. In cell culture, disulfiram was found to be a potent activator of autophagy, the malfunctioning of which has negative consequences on metabolic regulation. Thus, repurposing disulfiram may represent a potent strategy to combat obesity.

## Introduction

The challenges posed by the onslaught of obesity and associated metabolic disorders in the realm of public health and global economies are growing at a fast pace. Current behavioral and pharmacotherapies to reduce appetite and counter body weight gain have shown limited success in part due to resilience of the organism to adapt to changes in energy homeostasis^1,2^. Effective approaches for weight loss programs require negative energy balance where energy expenditure (physical activity and thermogenesis) exceeds calorie intake. Under conditions of high energetic demand, there is a close association between food intake and the amount of energy being used. However, as energetic needs are diminished, this link is lost leading to obesity and metabolic dysfunction^3,4^. Although lifestyle changes can help to restore energy balance, for many individuals this approach is not sufficient or sustainable long term. As such, new approaches to help raise resting metabolic rate and restore metabolic homeostasis will be necessary to help combat this growing public health crisis.

Disulfiram (DSF, Antabuse®), a Food and Drug Administration-approved alcohol aversive drug, has anti-inflammatory properties and confers protection from a number of cancer types^5^, in part through its inhibition of nuclear factor-κB (NF-κB)^6^. Subsequent work demonstrated that DSF can also regulate FOXO1, leading to impaired hepatic gluconeogenesis^7^, suggesting it may be involved in hepatic regulation of metabolic function. We recently reported that C57BL/6J mice on high-fat diet (HFD) responded to DSF treatment with a decrease in body weight and an increase in energy expenditure without a significant change in food intake^8^. In contrast to the leptin sensitizer celastrol^9^ and the incretin-dexamethasone conjugate^10^, DSF did not induce hypophagia. The intervention with DSF improved glucose tolerance and insulin sensitivity, while preventing fibrosis and steatosis of the liver and hypertrophy of pancreatic islet cells. The DSF’s catabolic actions were associated with loss of fat mass and lower circulating leptin levels in mice fed either standard diet (SD) or HFD, consistent with the known association between plasma leptin, weight gain, and insulin resistance^11,12^. The in vivo effect in the DSF intervention study demonstrated a ~40% reduction in weight in HFD-fed mice^8^. This is easily on par with or even superior to celastrol and incretin-based pharmacotherapy, which prompted a reduction in body weight in the ~25% range in obese mice^10,13,14^.

In our previous study, we also showed that DSF treatment elicited a substantial positive impact on health markers in the diabetes-prone Sprague–Dawley rats, with a key reduction in fat depots. This significant fat mass loss was associated with a trend toward lower accumulation of liver triglycerides in response to DSF. Moreover, at the conclusion of the 12-week study, the impact of DSF on the size and frequency of liver sinusoidal endothelial fenestrations^15^ was consistent with DSF’s ability to improve organismal response through preservation of hepatic function^8^. To gain insight into the basis for the health benefits of DSF, we performed liver transcriptomic and proteomic analyses, as well as serum metabolomic profiling in the same cohort of animals. This multi-omic approach allowed us to identify consistent signatures in response to DSF, including the identification of numerous pathways related to lipid and energy metabolism, redox, and detoxification. Gene Ontology (GO) analysis in the liver of DSF-treated mice returned the term “lysosomes,” the catalytic component in autophagy, among the top terms and cellular pathways. This along with the systemic anti-inflammatory effects of DSF led us to further explore the role of autophagy in the maintenance of cellular and organismal energy homeostasis^16^, as different variants of autophagy are activated in response to nutritional challenges, including nutrient deprivation and lipid excess. Activation of autophagy protects against lipotoxicity^17,18^, whereas sustained dietary lipid challenges lead to autophagy malfunction with the subsequent metabolic deregulation^19,20^. Our in vitro findings demonstrate that DSF has a direct effect on autophagy, indicating that this may be an important mechanism by which DSF regulates obesity and metabolic function.

## Results and discussion

### Transcriptional profiling of the effect of DSF in mouse liver

**Fig. 1:**
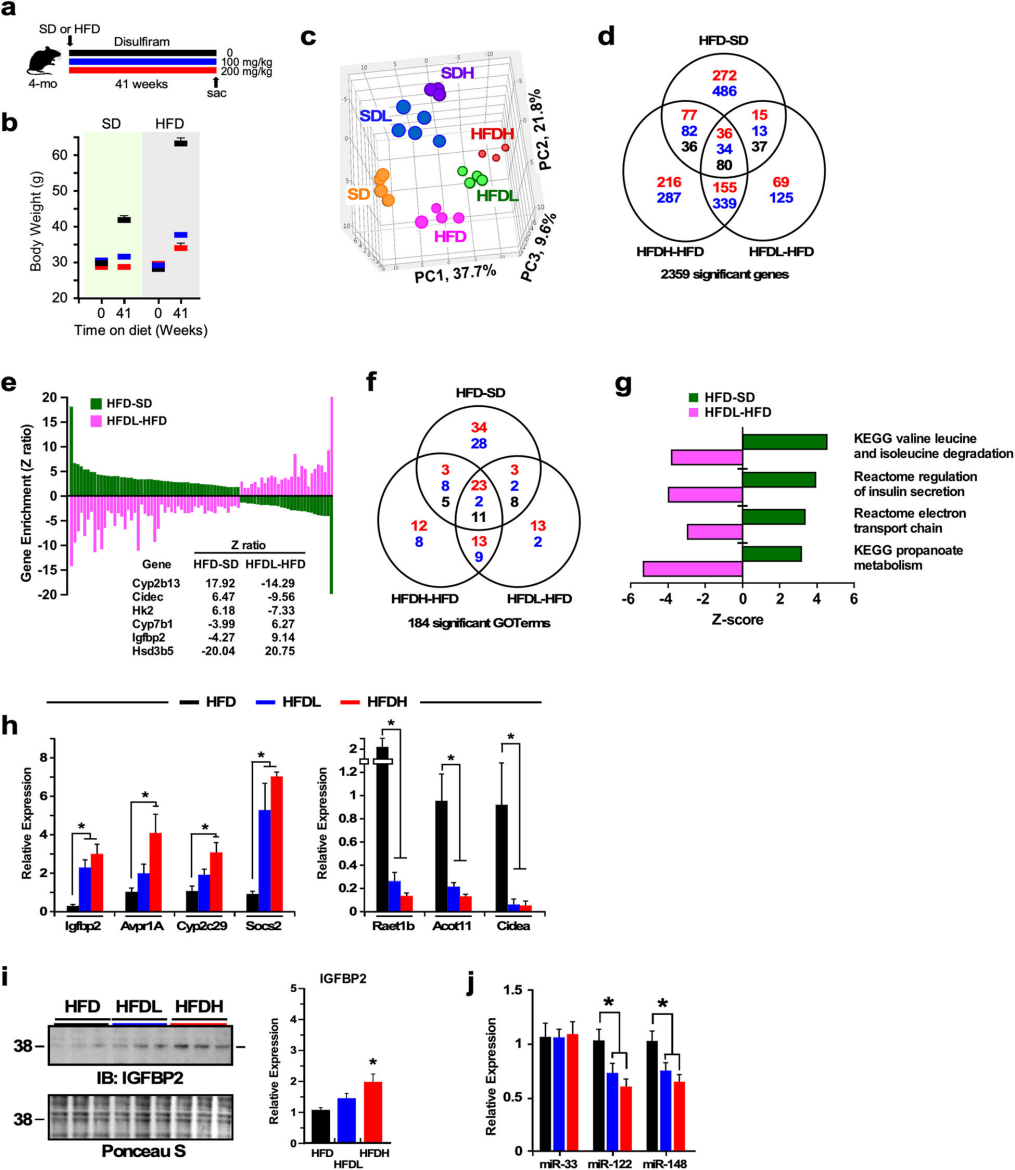
Disulfiram significantly modifies the liver transcriptome profile in HFD-fed mice. **a** Experimental design. Vehicle, black bar; low dose of DSF, blue bar; high dose of DSF, red bar. **b** Body weight of the six experimental groups of animals at the start and the conclusion of the study. *n* = 14–22 per group. **c** Principal component analysis (PCA) was performed on liver of mice fed a standard (SD) or high-fat diet (HFD) supplemented or not with low and high doses of DSF for 41 weeks. **d** Venn diagram of significantly upregulated (red font), downregulated (blue font), and reciprocally regulated (black font) gene transcripts. **e** Graphical representation of the 80 genes reciprocally regulated in the HFD-SD, HFDL-HFD, and HFDH-HFD (data not shown) pairwise comparisons. *Cyp2b13*, *Cidec*, *Hk2*, *Cyp7b1*, *Igfbp2*, and *Hsd3b5* were among the top reciprocally regulated liver genes. Additional information is provided in Supplemental Table 3. **f** Venn diagram depicting the distribution of GO Terms with positive (red font) and negative (blue font) z-ratios derived from the HFD-SD, HFDL-HFD, and HFDH-HFD pairwise comparisons. The number of GO Terms in black represents z-ratios in opposite direction between the three pairwise comparisons. **g** A select group of canonical pathways enriched in genes significantly impacted in the HFD-SD and HFDL-HFD pairwise comparisons. **h** Validation of the microarray data by quantitative real-time PCR. *n* = 4. **i** Liver extracts were prepared from mice after 41 weeks of dietary intervention and then immunoblotted for IGFBP2 (left panel). Relative protein expression after data normalization using Ponceau S staining of the membrane is depicted in right panel. Data in **f**, **g** are shown as mean ± SEM. *P ≤ 0.05 compared with diet without DSF. Related to Supplementary Fig. 1 and Supplementary Tables 1–3 and 6.

Four-month-old male C57BL/6J mice were fed either a SD or HFD supplemented with or without a low or high dose of DSF for 41 weeks (Fig. 1a). At the conclusion of the study, the body weight of the DSF-treated mice was significantly lower than their respective SD and HFD controls (Fig. 1b), even though mice were consuming similar amount of food. Additional information can be found in the original publication^8^.

The anti-obesity effect of DSF led us to perform whole-genome microarray analysis on mouse liver samples to identify and characterize the pathways and genes impacted by DSF. Principal component analysis (PCA), displayed as a three-dimensional scatter plot, helped visualize the impact of diet and doses of DSF on global gene expression profiles (*n* = 3–5 biological replicates/experimental group) and showed a strong effect of both diet (PC1 = 37.7%) and doses of DSF (PC2 = 21.8%) on the liver transcriptome (Fig. 1c). Venn diagrams were built to analyze lists of genes and visualize their intersections from various pairwise comparisons (Fig. 1d and Supplementary Fig. 1a). Pairwise comparisons of HFD- vs. SD-fed mice, and HFD-fed mice treated with either a low (HFDL, 100 mg/kg food/day) or high dose (HFDH, 200 mg/kg food/day) of DSF vs. HFD identified 2359 differentially expressed genes (Fig. 1d). Among these, there were 150 shared transcripts between all three comparisons, of which >53.3% (80/150) exhibited a reciprocal pattern of expression between HFD feeding and DSF treatment (Fig. 1e), with *Cyp2b13*, *Cidec*, *Hk2*, *Cyp7b1*, *Igfbp2*, and *Hsd3b5* being among the top reciprocally regulated genes (additional information can be found in Supplementary Table 1). In SD-fed mice, DSF treatment altered the expression of more than 1526 genes, with 677 of them shared between the SDL-SD and SDH-SD comparisons (Supplementary Fig. 1a and Supplementary Table 1 for a list of top regulated genes). Parametric analysis of gene-set enrichment (PAGE) enables the identification of canonical pathways and unbiased GO annotations enriched in genes present in various pairwise comparisons. There were 102 significantly enriched GO Terms in the SDL-SD and SDH-SD pairwise comparisons (Supplementary Fig. 1b), of which 54 (52.9%) intersected and exhibited a similar pattern of expression (Supplementary Table 2). In addition, 62 out of a total of 173 canonical pathways (35.8%) were shared between SDL and SDH vs. SD (Supplementary Fig. 1b and Supplementary Table 2). Top upregulated pathways included “reactome glutathione conjugation,” “KEGG ribosome,” and “KEGG metabolism of xenobiotics by CYP450,” while “Reactome hormone-sensitive lipase-mediated triglyceride hydrolysis” was among the top downregulated pathways (Supplementary Fig. 1c). Further analysis revealed that 184 GO Terms were significantly enriched in the HFD-SD, HFDL-HFD, and HFDH-HFD pairwise comparisons, of which 36 intersected and 30.6% of these (11/36) exhibited a reciprocal pattern of expression (Fig. 1f and Supplementary Table 2), including “KEGG valine leucine and isoleucine degradation” and “Reactome electron transport chain” (Fig. 1g). Additional information on DSF-responsive GO terms and canonical pathways in HFD-fed mice can be found in Supplementary Table 2.

Four-way Venn diagrams were constructed to identify overlapping genes and genes that were uniquely expressed in the HFDH-HFD, HFDL-HFD, SDH-SD, and SDL-SD pairwise comparisons. In response to DSF, more than 65 overlapping genes were upregulated and 81 downregulated irrespective of the diet (Supplementary Table 3). Six CYP genes were significantly altered by DSF treatment, which included three members of the *Cyp2c* subfamily that are known to dampen hepatic inflammatory processes (reviewed in ref. ^21^). The overlapping genes that were the most highly upregulated included *Igfbp2* and *Avpr1a*, while *Cidea*, *Raet1b*, and *Acot11* were among the top downregulated genes. Quantitative reverse-transcription PCR was used to confirm the gene expression results from the microarray analysis (Fig. 1h and Supplementary Fig. 1d). Compared with SD-fed controls, high-dose DSF treatment upregulated hepatic insulin-like growth factor binding protein 2 (IGFBP2) content regardless of the diet (Fig. 1i and Supplementary Fig. 1e), supporting the notion that IGFBP2 is independently associated with protection from HFD-induced obesity and confers increased hepatic insulin sensitivity^22,23^.

Hence, the ability of DSF to counteract obesity and insulin resistance while eliciting anti-inflammatory signaling was associated with marked upregulation of several hepatic genes, including *Igfbp2*, *V1aR*, and *Cyp2c*^22,24–27^. We also observed downregulation of both *Cidea*, a fatty acid-induced transcriptional coactivator^28^, which contributes to diet-induced obesity and diabetes^29^, and *Acot11*, which encodes a medium/long-chain acyl-CoA thioesterase whose induction is implicated in hepatic glucose production and insulin resistance^30^.

This data highlights a striking molecular signature in the liver of DSF-treated mice consistent with protection from metabolic dysregulation and inflammation both under a normal and an obesogenic environment.

### DSF reduces hepatic oxidative stress

**Fig. 2:**
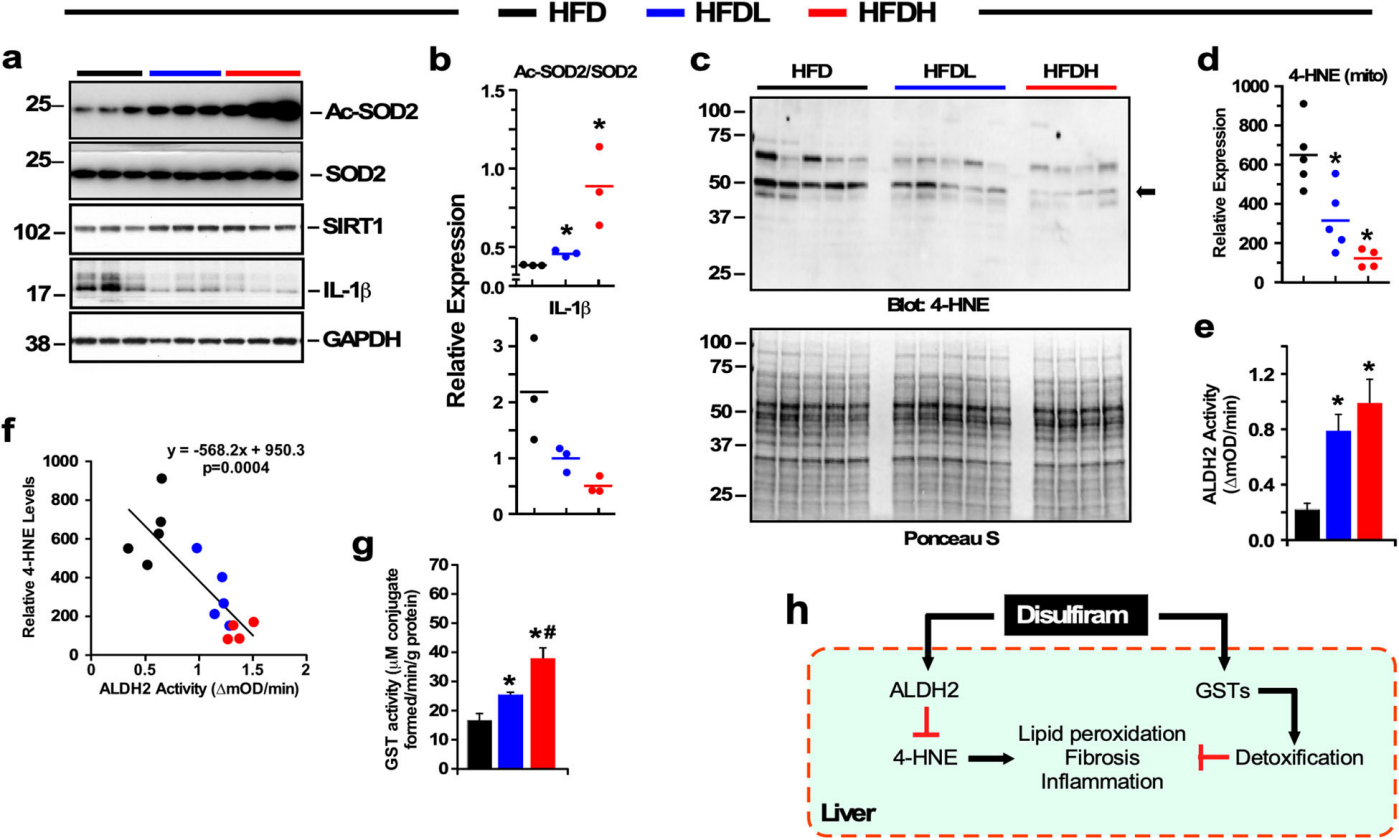
DSF reduces oxidative stress and acetylation markers in HFD-fed mice. **a** Detection of acetylated and total forms of SOD2, SIRT1, and IL-1β proteins in liver homogenates. **b** Relative protein expression (acetylated/total SOD2 ratio and IL-1β) after data normalization using GAPDH as loading control. **c** Detection of 4-HNE-conjugated proteins in liver homogenates. Ponceau S staining of the membrane confirmed equal protein load (lower panel). **d** Densitometric analysis of 4-HNE signals after normalization with Ponceau S. Data are shown as mean ± SEM. *P ≤ 0.05 compared with diet without DSF; ^#^*P* ≤ 0.05 compared with low DSF. Related to Supplementary Fig. 2.

DSF may have beneficial effects on hepatic function by evoking effective protection against activation of cellular pro-inflammatory processes. Immunoblotting of mouse liver extracts revealed that DSF treatment significantly reduced the accumulation of the Lys-68 acetylated form of superoxide dismutase 2 (SOD2) independent of diet (Fig. 2a, b and Supplementary Fig. 2a, b). SOD2 deacetylation is associated with increased mitochondrial antioxidant activity^31^, which improves mitochondrial ROS homeostasis and autophagic flux^32,33^. DSF treatment also reduced the levels of interleukin (IL)-1β (Fig. 2a, b) and the phosphorylated, active form of p65Rel protein—encoding for the transactivating subunit of NF-κB—(Supplementary Fig. 2a, b) in HFD and SD livers, respectively.

DSF treatment significantly reduced 4-HNE levels (Fig. 2c, d Supplementary Fig. 2c, d), while inducing cytosolic glutathione-*S*-transferase (GST) activity in the livers of mice fed either HFD or SD (Fig. 2e and Supplementary Fig. 2e), consistent with the detoxification of peroxidized lipids and other electrophilic xenobiotics.

### Impact of DSF on the rat liver proteome

**Fig. 3:**
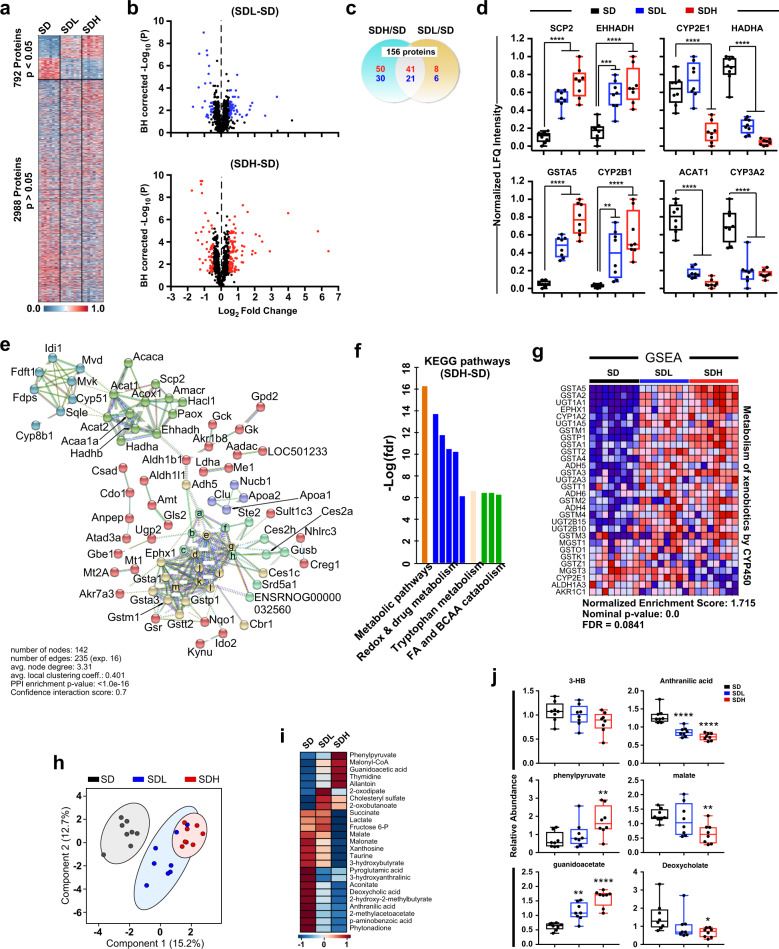
Multi-omics analysis of the effects of disulfiram in rats. Proteomic analysis of liver proteins (**a**–**g**) and untargeted serum metabolomics (**h**–**j**) in rats exposed to disulfiram. **a** Heat map visualization of protein abundance in livers of rats fed laboratory SD (control) either supplemented or not with a low or high dose of DSF. Upregulation (red font), downregulation (blue font). *n* = 8 per group. **b** Volcano plots of liver protein abundance changes after diet supplementation with low or high dose of DSF were plotted with the *y*-axis showing the Benjamini–Hochberg corrected −log10 (*P*) and the *x*-axis showing the log2 fold change of protein abundance (DSF/control SD) calculated from the median LFQ intensity values. The blue and red symbols denote significant changes with low and high DSF, respectively, and the black symbols denote nonsignificant changes. Significance is defined as >40% median fold changes in either direction. **c** Two-way Venn diagram depicting the distribution of unique and common proteins whose expression was impacted by DSF (low or high dose) vs. control SD. Upregulation (red font), downregulation (blue font). Significance is defined as >40% median fold changes in either direction. **d** Abundance of a select group of proteins significantly impacted by DSF treatment. Normalized LFQ intensity values are represented in box and whisker plot format (*n* = 8 per group). Statistics for the effects of DSF intervention represent the *p*-value from a one-way ANOVA with Dunnett’s post hoc tests. *P < 0.05, **P < 0.01, ***P < 0.001, and ****P < 0.0001. **e** Clustering of liver proteins significantly impacted by DSF treatment as provided by String protein–protein interaction database. High confidence interaction score was selected (0.7). For reasons of clarity, some of the proteins present in the redox & drug metabolism cluster were labeled ‘a’ to ‘m’. These are: a, Aldh1a1; b, Ugdh; c, Cyp2b1; d, Cyp2e1; e, Cyp4a1; f, Cyp3a1; g, Ugt1a1; h, Ugt2b1; i, Cyp2c6v1; j, Cyp2c13; k, Cyp1a2; l, Cyp2c12; m, Gsta2. **f** Top enriched ten pathways generated from these experimental and predicted interactions map for “metabolic pathways,” “Redox and drug metabolism,” Tryptophan metabolism,” and “fatty acid (FA) and branched-chain amino acid (BCAA) catabolism.” **g** Gene-set enrichment analysis (GSEA) depicting a set of proteins involved in “metabolism of xenobiotics by CYP450” whose expression was significantly impacted by DSF supplementation. **h** PLS-DA of untargeted serum metabolomics (*n* = 8 in each group). **i** Heat map **i**llustrates the relative average of each metabolite contributing to the group separation between control and DSF (low and high). **j** Relative abundance of a select group of metabolites. The data are represented in box and whisker plot format. *P < 0.05; **P < 0.01; ****P < 0.0001 vs. control group. Related to Supplementary Fig. 3 and Supplementary Tables 4 and 5.

The major role the liver has on regulation of whole-body metabolism led us to assess the changes induced by DSF in the liver proteome. To achieve this goal, 3-month-old male Sprague–Dawley rats, identified as prone to the development of obesity, were maintained for 12 weeks on either standard SD alone or SD supplemented with low and high doses of DSF (100 and 200 mg/kg Body Weight (BW)). A heat map of the untargeted label-free quantification (LFQ) for all 3780 proteins ranked by fold change is depicted in Fig. 3a. Of these, 792 proteins had a Benjamini–Hochberg (BH)-corrected *P*-value < 0.05 without consideration of fold changes between low or high dose of DSF vs. control SD. Volcano plots were then generated to visualize the statistically significant differences in response to the DSF supplementation, identified by arbitrarily setting median fold changes at 40% in either direction (Fig. 3b). This led to the generation of a Venn diagram representing the distribution of 156 proteins that were unique and shared among the high DSF-control and low DSF-control pairwise comparisons (Fig. 3c). Of these DSF-shared proteins, GST α5 and cytochrome P450 2B1 were among the most significantly upregulated, while cytochrome P450 3A2 and mitochondrial acetyl-CoA acetyltransferase (ACAT1), long-chain 3-hydroxyacyl-CoA dehydrogenase and 3-ketoacyl-CoA thiolase were among the most significantly downregulated (Supplementary Table 4). A strong positive correlation in the expression level of statistically significant proteins between low and high DSF was found (Fig. 3a), with DSF eliciting a dose-dependent effect on protein abundance. The normalized LFQ intensity for several proteins with functional associations and/or relationships with key biological pathways confirmed this pattern (Fig. 3d and Supplementary Fig. 3a). As the overall function of a given protein is frequently defined by its protein interactors, we used the curated 156 protein input and employed STRING functional network association program (https://string-db.org/) to investigate the strength of interactions between the proteins that were co-expressed in response to DSF; a high strength reliability cut-off (0.7) was applied and all unconnected nodes were removed from the network (Fig. 3e). This network demonstrated a very high enrichment probability (PPI, *P* < 1 × 10^–16^). Unbiased *k*-means clustering was employed to group the interacting proteins in the network into five main clusters that were associated with: (i) xenobiotic and unsaturated fatty acid metabolic process (yellow); (ii) fatty acid catabolic process (green); (iii) drug metabolic process (aqua marine); (iv) sterol biosynthetic process (dark cyan); and (v) reverse cholesterol transport (purple) (Fig. 3e). Reinforcing the unbiased nature of the known molecular functionalities of this interactome, this metadataset was further analyzed for biological pathways using the KEGG module database. We found that similar functions (i.e., metabolic pathways, redox and drug metabolism, and fatty acid/branched-chain amino acid catabolism) were present among the most enriched signaling networks following DSF intervention (Fig. 3f and Supplementary Table 5). Lastly, Gene-set enrichment analysis^34^ of the rat liver proteome was performed. This indicated that defined sets of proteins involved in nine KEGG pathways showed statistically significant differences between low and high DSF vs. control group (Supplementary Table 5), and were reminiscent of those identified using STRING database. Heatmaps showing the abundance of each protein present within three selected KEGG pathways, namely “metabolism of xenobiotics by CYP450” (Fig. 3g) as well as “peroxisome” and “PPAR signaling pathway” (Supplementary Fig. 3b), are depicted for each individual rat liver of control and low/high DSF supplemented groups.

### Impact of DSF on the rat serum metabolome

The reduced adiposity that we previously observed in DSF-treated rats^8^ likely resulted, in part, from substantial changes in whole-body metabolism through WAT reprogramming. Combining univariate (one-way analysis of variance (ANOVA)) and multivariate (partial least square discriminant) statistical analyses of serum metabolomes from the three experimental groups of rats enabled us to obtain a subset of 25 metabolites (out of 69) that accounts for the separation between the metabolite profiles acquired for SD alone or SD supplemented with low and high dose of DSF. This approach displayed a sharp separation between the control group and the two DSF groups, with the latter groups showing considerable overlap (Fig. 3h).

We next generated heatmaps depicting both the 25 metabolites that contributed to the separation between the three experimental groups (Supplementary Fig. 3c) and average signals for each group (Fig. 3i). Compared with SD controls, the relative levels of malonyl-CoA, an inhibitor of carnitine palmitoyl transferase 1, were dose-dependently increased as a function of DSF concentration. This is in tune with the decrease in 3-hydroxybutyrate (3-HB) in response to DSF, which would mean that less lipids are being transported and utilized by mitochondria. This in turn links with the decreases in aconitate, succinate and malate, three main metabolites from the Tricarboxylic Acid (TCA) cycle. Metabolites from several lipid-related pathways were negatively impacted by DSF, including malonate (fatty acid synthesis), 2-hydroxy-2-methylbutyrate and 2-methylacetoacetate (FA metabolism and ketone bodies), and deoxycholate (secondary bile acid metabolism). Allantoin is an end product of adenine nucleotide degradation that appears to be increased with DSF treatment, while the abundance of xanthosine was markedly reduced, consistent with purine nucleotide catabolism. Interestingly, the depletion of the tryptophan metabolites anthranilic acid and 3-hydroxyanthranilic acid (3-HAA) supports a systemic anti-inflammatory role of DSF. The profile of select metabolites is depicted in Fig. 3j.

The ability of DSF to influence the hepatic tryptophan/kynurenine pathway may represent an alternative mechanism to combat diet-induced insulin resistance^35^. Data from an earlier work showed that DSF administration in rats allows for the accumulation of serum 3-HAA^36^. Here we observed a significant reduction in tryptophan metabolites, anthranilic acid, and 3-HAA, in serum of DSF-treated rats together with altered expression of two key enzymes implicated in hepatic tryptophan catabolism, IDO2 and KYNU, with the latter being involved in the kynurenine pathway for NAD biosynthesis and redox reactions^37^.

Thus, a combination of ‘omics’ analyses were conducted in liver extracts of mice and rats and rat serum to provide insight into the underlying biochemical changes arising from DSF signaling. Integrated application of transcriptomics of mouse liver and proteomic response in rat liver yielded molecular signatures consistent with altered pathways related to “lipid and energy metabolism,” “redox, xenobiotics and drug metabolic process,” and “peroxisome” among others. The reduced amount of hepatic ACAT1 protein, the last step in fatty acid β-oxidation, coincided with the depletion of 3-HB and other lipid-related metabolites, as well as metabolites from the TCA cycle in serum of DSF-treated rats.

### DSF activates cellular autophagy

As indicated above, treatment with DSF markedly reduced markers of lipid peroxidation in the liver, while increasing the activity of the detoxification enzyme, GST. Combatting hepatic inflammation and oxidative stress through regulation of autophagy offers an attractive therapeutic strategy. Autophagy is an evolutionarily conserved process that contributes to turnover of intracellular proteins and organelles and the management of stress responses. In light of the relevance of autophagy in metabolism and the fact that GO analysis of DSF-treated animals returned the term “lysosomes,” the catalytic compartment in autophagy, among the top terms and cellular pathways (Supplementary Table 2), we were motivated to explore whether DSF had a direct effect on autophagy. To achieve this goal, we utilized a standard cell culture system with murine NIH3T3 fibroblasts where autophagy and its different variants have been well characterized^38–40^.

**Fig. 4:**
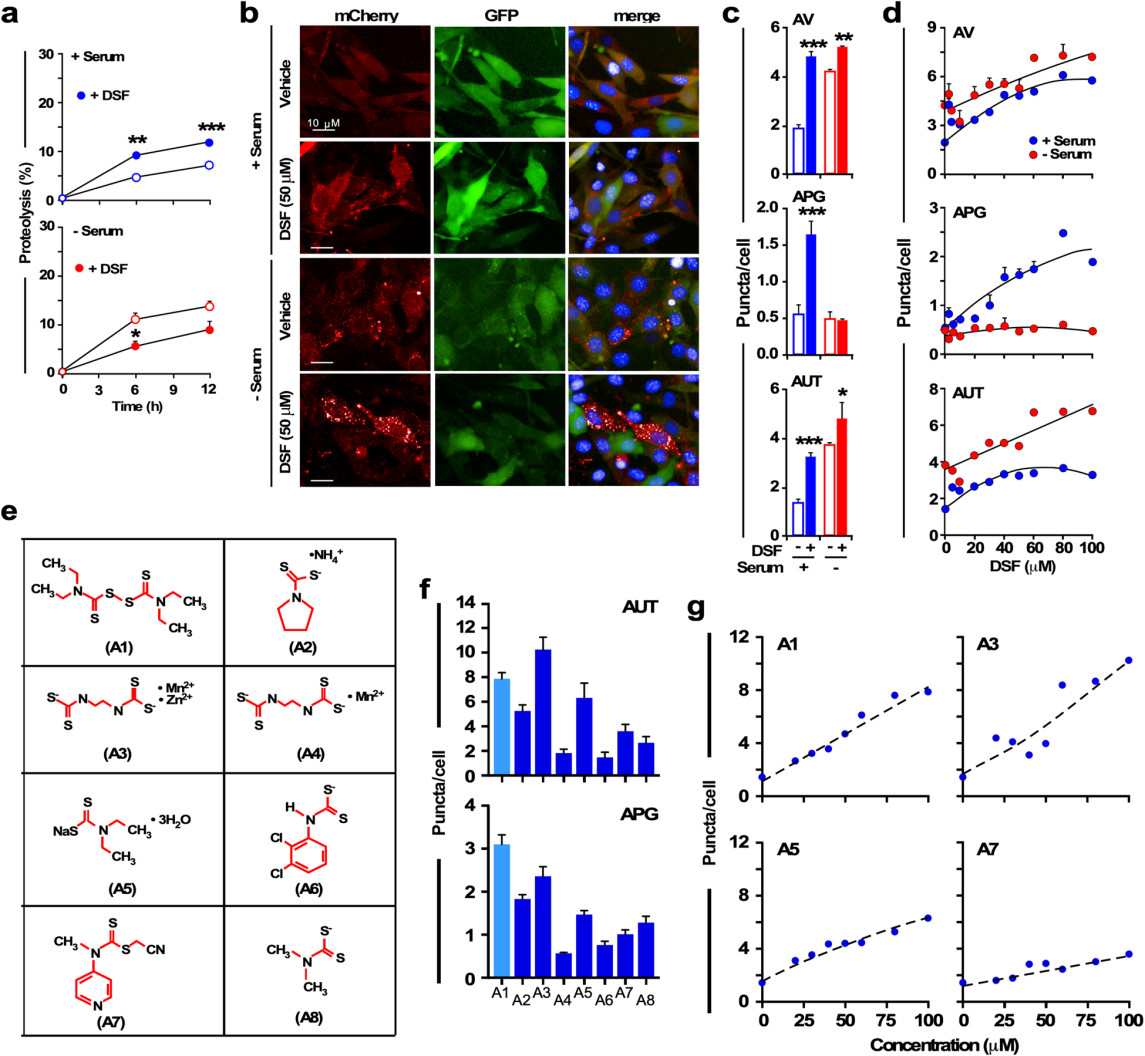
Disulfiram activates autophagy in cultured cells. **a** Effect of disulfiram (DSF) on the degradation of long-lived proteins. Murine NIH3T3 fibroblasts in culture were labeled with ^3^H-leucine for 48 h and incubated in complete (serum+) or serum-free medium in the presence of 20 μM DSF or not. Rate of proteolysis was calculated as the percentage of the initial acid-precipitable radioactivity (proteins) transformed into acid-soluble radioactivity (amino acids and small peptides) at the indicated times. All values are mean ± SEM of three independent experiments, each performed with triplicate wells per time point. There is no error bar in the top panel because the error bar is shorter than the size of the symbol. *P < 0.05; **P < 0.01; ***P < 0.001 vs. without DSF. **b–d** Effect of DSF on basal and inducible autophagy. NIH3T3 cells expressing the tandem reporter mCherry-GFP-LC3 were exposed to 50 μM DSF for 24 h in complete (+Serum) or serum-free medium (−Serum). **b** Representative images of the individual and merged channels in cells where nuclei were highlighted by DAPI staining; **c** The number of autophagic vacuoles (AV), autophagosomes (APG), and autolysosomes (AUT) was determined by high-content microscopy. **d** Dose dependence of the activating effect of DSF on macroautophagy was analyzed in cells exposed to increasing concentrations of DSF. Number of AV (top panel), APG (middle panel), and AUT (bottom panel) was determined by high-content microscopy. **e**–**g** Structure activity of dithiocarbamate analogs on autophagy. **e** Structures of the eight compounds t**e**sted. **f** mCherry-GFP-LC3 reporter-expressing NIH3T3 cells were exposed for 24 h to 100 μM of DSF (A1) and DSF analogs (A2–A8) for the measure of AUT (top panel) or APG (bottom panel). **g** Changes in the number of AUT after exposure to increasing concentrations of A1, A3, A5, and A7. **c**–**g** Unless otherwise indicated, all values are mean ± SEM and quantifications were done in at least 2500 cells per condition in three different experiments using high-content microscopy. Abbreviations: A1, bis(diethylthiocarbamate) disulfide aka disulfiram; A2, ammonium pyrrolidine dithiocarbamate; A3, Mn^2+^-Zn^2+^ ethylenebis(dithiocarbamate); A4, Mn^2+^ ethylenebis(dithiocarbamate) aka pestanal; A5, Na^+^ diethyldithiocarbamate trihydrate; A6, triethylammonium *N*-(3,4-dichlorophenyl) dithiocarbamate; A7, *S*-cyanomethyl-*N*-methyl-*N*-(pyridin-4-yl) dithiocarbamate; A8, Zn^2+^ dimethyldithiocarbamate. Related to Supplementary Fig. 4.

Using a metabolic labeling to quantify intracellular protein degradation, we found that addition of DSF (20 μM) stimulated the degradation of long-lived proteins (common substrates for degradation by lysosomes) in cells maintained in serum-supplemented media. This effect was not observed upon serum removal, a condition known to already activate lysosomal protein degradation (Fig. 4a). Next, we directly analyzed the effect of DSF in macroautophagy, the best characterized type of autophagy, using high-content microscopy in cells expressing a tandem mCherry-GFP-LC3 reporter^41^ (Fig. 4b–d). This reporter highlights autophagosomes (APG) in both fluorescent colors, but because green fluorescent protein (GFP) fluorescence is quenched at low pH, autolysosomes (AUT) (resulting from fusion of APG with lysosomes) only fluoresce in red (Fig. 4b). Maturation of APG into AUT is a good measurement of autophagic flux. Addition of DSF (50 μM) under basal conditions (+Serum) significantly increased the total number of autophagic vacuoles due to a proportional increase in both APG and AUT, in support of an overall increase in the autophagic flux (Fig. 4c). As in the case of intracellular protein degradation, addition of DSF in cells grown in absence of serum had only a minor stimulatory effect on autophagy, in support of DSF sharing common autophagy-activating mechanisms with nutrient deprivation. Interestingly, the increase in APG number observed under basal conditions was no longer apparent upon serum removal, suggesting better capacity of lysosomes to rapidly degrade the forming APGs under these conditions (Fig. 4c). We confirmed that the DSF effect on autophagy was dose dependent (Fig. 4d), and that, once again, the stimulatory effect of DSF was higher on basal autophagy (+Serum). In fact, DSF was able to bring basal macroautophagy to similar levels as those observed in serum-free conditions (Fig. 4d). The dose-dependence study also confirmed that the differences in APG content in DSF-treated cells with and without serum were mostly due to more efficient APG clearance in the serum-deprived cells.

DSF is a member of the dithiocarbamate family of mono-anionic metal chelator agents whose anti-cancer activity^42^ may partly be explained by its ability to stimulate autophagy. A series of eight dithiocarbamate analogs with different side chains (Fig. 4e) were tested for their autophagic activity. Of these, Mn^2+^-Zn^2+^ ethylenebis(dithiocarbamate) (compound A3) and Na^+^ diethyldithiocarbamate trihydrate (compound A5) were as potent as DSF (compound A1) at promoting autophagy flux in a dose-dependent manner, whereas Zn^2+^ dimethyldithiocarbamate (compound A7) was representative of compounds with more modest autophagy-enhancing activity (Fig. 4f–i and Supplementary Fig. 4a, b).

Our study has shown that autophagy is one of the cellular targets by which DSF mediates its effects at the cellular level. In light of the tight relationship between nutritional state and autophagy, and the previously described inhibitory effect of HFD on autophagy^16,17^, the observed increase in autophagy flux upon treatment with DSF suggests that DSF may exert its beneficial effect in part by preventing the toxic effect of HFD on the autophagy system. Autophagy plays important roles in cellular quality control, maintenance of cellular homeostasis, and numerous other cellular processes. We surmise that autophagy malfunction contributes to the defective hepatic lipid and glucose metabolism, steatosis, and associated defects in proteostasis observed in response to HFD, and that the ability of DSF treatment to stimulate autophagy without nutrient deprivation may contribute to its beneficial effects on liver and whole-body metabolic function. Although we provided experimental evidence into the effect of DSF on autophagy in isolated fibroblasts, additional in vivo or in vitro studies using models of altered autophagy will be required to demonstrate the causality of this mechanism.

Autophagy is well known to be regulated by the mammalian target of rapamycin (mTORC1) and AMP-activated protein kinase (AMPK), two upstream nutrient and energy sensors^43,44^. Previous work in HepG2 cells has demonstrated the ability of pyrrolidine dithiocarbamate (a DSF analog) to inhibit mTORC1 through induction of DNA damage-inducible transcript 4 (DDIT4, also known as REDD1)^45^. In the current study, a clear increase in autophagy emerged in fibroblasts treated with pyrrolidine dithiocarbamate (compound A2, Fig. 4) and other members of this class of compounds, indicating a potential mechanism by which DSF regulates autophagy. Along those lines, it would have been interesting to have considered changes in AMPK activity, which will be the object of future investigations.

DSF confers protection from acetaminophen-induced liver damage^46^ by inhibiting cytochrome P450 2 E1 isoform (CYP2E1)^47,48^, an iron-donating catalyst implicated in the production of free radicals that damage key macromolecules (e.g., DNA, proteins, and lipids) leading to tissue injury and cell death. The ability of DSF to influence CYP expression and/or activity implies changes in CYP450-mediated metabolism of drugs and possibly other xenobiotics with unanticipated drug–drug interactions. Because of the variability in the pattern of genetic variation between different populations for both CYP450 isozymes and phase II drug-metabolizing enzymes^49,50^, quantitative changes in proteins encoding both classes of enzymes as a consequence of DSF supplementation may have potentially serious implications in predicting the fate and pharmacological consequences of xenobiotics and drugs. Accordingly, careful clinical drug interaction studies using a broad set of combinatorial approaches targeting supplements and frequently prescribed drugs in the management of blood cholesterol, blood pressure, diabetes, and other medical conditions must be conducted to assess the risk of adverse DSF toxicity. Overall, these findings support our previous work in demonstrating the high potential for repurposing DSF to treat patients with obesity and metabolic dysfunction.

## Methods

### Husbandry, diets, and dietary intervention

Liver and serum samples from mice and rats were collected from the same animals reported in Bernier et al.^8^. All animal protocols were approved by the Animal Care and Use Committee (444-TGB-2016) of the National Institute on Aging and the University of Sydney Animal Welfare Office, ethics number: 2018/1365.

#### Microarray analysis

Total RNA was isolated from mouse liver with Trizol® (Invitrogen) and then hybridized to BD-202-0202 Illumina BeadChips. Total RNA was quality-controlled using the Agilent Bioanalyzer RNA 6000 Chip (Agilent) and labeled according to the manufacturer’s instructions using the Illumina® TotalPrepTM RNA amplification kit. A total of 750 ng biotinylated aRNA was hybridized to mouse Ref-8v 2BeadChips (Illumina). Following posthybridization rinses, arrays were incubated with streptavidin conjugated Cy3 and scanned using an Illumina BeadStation 500X Genetic Analysis Systems scanner. Hybridization intensity data were extracted from the scanned images using Illumina BeadStudio GenomeStudio software, V2011.1. Raw data were subjected to *Z*-normalization, as described elsewhere^51,52^. PCA was performed on the normalized *Z*-scores of all of the detectable probes in the samples. Significant genes were selected by the *z*-test < 0.05, false discovery rate < 0.30, as well as *z*-ratio > 1.5 in both directions and ANOVA *P*-value < 0.05. PAGE was analyzed as previously described^53^; *n* = 5 per group; age = 57 weeks; diet = 41 weeks.

#### Quantitative real-time PCR

Total RNA was isolated from the liver using the Trizol reagent (Invitrogen). Complementary DNA was reverse transcribed using the High Capacity cDNA reverse-transcription kit (Applied Biosystems). The real-time PCR was performed on individual cDNAs by using SYBR® Green PCR master mix in StepOne plus Real-time PCR system (Applied Biosystems) to measure duplex DNA formation. The calculation of mRNA expression was performed by the 2^−ΔΔCT^ method normalized to the expression of Hprt and 18S. The oligonucleotide primer sequences are found in Supplementary Table 6; *n* = 5 per group; age = 57 weeks; diet = 41 weeks.

#### Metabolomic analysis of rat serum

Liquid chromatography–mass spectrometry (LC-MS) experiments were performed on an Agilent 1260 Infinity HPLC system (Agilent Technologies, Santa Clara, CA, USA) coupled to a tandem MS (MS/MS) with a triple quadrupole (QqQ) mass analyzer operating in MRM scan mode on the AB SCIEX QTRAP^®^ 5500 MS system. The separation of highly polar analytes such as citric acid cycle intermediates, nucleotide and nucleoside phosphates, high-energy intermediates, organic acids, and glycolytic intermediates, which ionize in the negative ionization mode, was achieved on a polar XBridgeAmide^TM^ column (Waters).^54^ As indicated by Hatchwell et al.^54^, all raw data files (Analyst software, version 1.6.2; AB Sciex, Foster City, CA, USA) were imported into the analysis software Multi-Quant 3.0 for MRM Q1/Q3 peak integration and data were normalized relative to pooled plasma samples that were analyzed in the sample queue after every 6 study samples. The value of each area corresponds to the abundance of that metabolite. To account for any performance drift in the LC-MS/MS, the metabolite abundance was normalized in each sample to the bookended pool plasma sample, deriving a “Normalized area” (normalized abundance) for each metabolite.”

### Proteomics analysis of rat liver

#### Reagents

Acetonitrile (Optima grade), isopropanol (Optima grade), water (Optima grade), methanol LC grade, ammonium hydroxide, formic acid, and TCEP were from Thermo Fisher Scientific (MA, USA). Trifluoroacetic acid (TFA), sodium deoxycholate, formic acid, chloroacetamide, and proteomics-grade trypsin were from Sigma-Aldrich (MO, USA). Ethyl acetate LC-MS grade was from Millipore (MA, USA). All other reagents were from Sigma-Aldrich.

### Liver sample preparation and cleanup using SDB-RPS stagetips

Liver sample preparation was performed as followed: briefly, 25 mg of frozen liver tissue was added to 1 mL of tissue homogenization buffer (4% sodium deoxycholate, 100 mM Tris-HCl pH 8.5) in an 8 mL tube at room temperature followed by immediate homogenization using an Ultra-Turrax T8 stick homogenizer (IKA®-Werke) for 10 s. Each sample was immediately transferred into a 2 mL tube and heated to 95 °C for 10 min. Samples were then sonicated for 10 min total time at 80% amplitude in a QSonica 800R2 instrument at room temperature (RT). Tubes were centrifuged at 18,000 × *g* for 10 min at RT and clarified lysates (~800 μL) were transferred into clean 2 mL tubes and stored at −20 °C. Protein determination was performed using the BCA total protein assay (Pierce, Thermo Fisher Scientific), which showed most extracts at 5 mg/mL.

Before proceeding with enzymatic digestion of the lysates, samples were thawed at 65 °C for 10 min with vortexing at 1000 r.p.m. on a ThermoMixer-C (Eppendorf). Protein (10 μg) was transferred to Protein Lo-bind 1.5 mL tubes (Eppendorf) and SDC buffer (4% sodium deoxycholate, 10 mM TCEP, 40 mM chloroacetamide, and 100 mM Tris-HCl pH 8.5) added (~23 μL) to a final volume of 25 μL. Samples were heated to 95 °C for 10 min at 1500 r.p.m. in an Eppendorf Thermomixer-C with a ThermoTop (heated lid) to denature, reduce, and alkylate proteins. Tubes were then centrifuged at 6000 × *g* for 1 min and cooled to RT prior to the addition of 75 μL of milliQ water (3-fold dilution) and 200 ng trypsin to each tube. Proteins were digested at 37 °C for 16 h at 1500 r.p.m. in a Thermomixer-C with a ThermoTop (heated lid). An equal volume (100 μL) of 99% ethyl acetate/1% TFA was added to the digested peptides to stop the digestion and solubilize the sodium deoxycholate with phase separation prior to SDB-RPS cleanup.

SDB-RPS StageTips were generated by punching double-stacked SDB-RPS discs (Sigma, catalog number 66886-U) with an 18-gauge needle and mounted in 200 μL tips (Eppendorf), as described previously^55^. For StageTip SPE processing using the Spin96, StageTips were inserted into a holder and placed in the top, which was then stacked onto the washbottom containing a polypropylene 96-well microtiter plate to collect the sample flowthrough and washes. Each tip was wetted with 100 μL of 100% acetonitrile and centrifuged at 1000 × *g* for 1 min. Following wetting, each StageTip was equilibrated with 100 μL of 0.1% TFA in H_2_O and 30% methanol/1% TFA with centrifugation for each at 1000 × *g* for 3 min^55^. A second equilibration step was performed with 100 μL of 0.2% TFA in H_2_O with centrifugation at 1000 × *g* for 3 min. Each StageTip was then loaded with the equivalent of ∼10 μg peptides by adding the entire lower aqueous phase with centrifugation at 1000 × *g* for 3 min. The peptides were washed twice with 100 μL of 99% ethyl acetate/1% TFA, which was followed by one wash with 100 μL of 0.2% TFA in water. For elution of peptides, the StageTips were mounted over a clean 96-well PCR plate. To elute, 100 μL of 5% ammonium hydroxide/80% acetonitrile was added to each tip and centrifuged as above for 5 min. Samples in the PCR plate were dried using a GeneVac EZ-2 using the ammonia setting at 35 °C for 60–75 min total drying time. Dried peptides were resuspended in 60 μL of 5% formic acid. Samples were stored at 4 °C until analyzed by LC-MS/MS.

### LC-MS/MS and analysis of spectra

Using a Thermo Dionex RSLCnano, 500 ng of peptides in 5% (v/v) formic acid (injection volume 3 μL) was directly injected onto a 75 μm × 50 cm fused silica column with a ~10 μm pulled tip packed with C18AQ (Dr. Maisch, Ammerbuch, Germany, 1.9 μm). As indicated by Harney et al.^55^ the column was coupled online to a nanospray ESI source. Peptides were resolved over gradient from 5% acetonitrile to 40% acetonitrile over 120 min with a flow rate of 300 nL min^−1^ at 60 °C. Peptides were ionized by electrospray ionization at 2.3 kV. MS/MS analysis was carried out on a Q-Exactive HFX mass spectrometer (Thermo) using Higher-energy collisional dissociation (HCD) fragmentation in positive mode. The data-dependent acquisition method used acquired MS/MS spectra of the top 20 most abundant ions at any one point during the gradient. MS1 scans were acquired from 300 to 1650 *m*/*z* (60,000 resolution, 3e10^6^ AGC target, 20 ms maximum injection time) and MS2 scans having a fixed first m/z of 140 (15,000 resolution, 1e10^5^ AGC target, 25 ms maximum injection time, 27 NCE, 1.4 *m*/*z* isolation width). RAW MS data have been deposited to the ProteomeXchange Consortium (http://proteomecentral.proteomexchange.org) via the PRIDE partner repository with the data set identifier PXD016793, username: reviewer58131@ebi.ac.uk, password: U2nPrkRn.

RAW data were analyzed using the quantitative proteomics software MaxQuant^56^ (http://www.maxquant.org, version 1.6.3.4) and the MaxQuant output has also been uploaded to the ProteomeXchange Consortium under the same identifier. This version of MaxQuant includes an integrated search engine, Andromeda^57^. Peptide and protein level identification were both set to a false discovery rate of 1% using a target-decoy based strategy, and proteins were filtered such that they must have >2 razor and unique peptides. The database supplied to the search engine for peptide identification contained both the rat UniProt database downloaded on the 1 April 2019, containing 31,553 protein sequence entries and the MaxQuant contaminants database. As indicated by Hatchwell et al.^54^, mass tolerance was set to 4.5 p.p.m. for precursor ions and MS/MS mass tolerance was 20 p.p.m. Enzyme specificity was set to trypsin (cleavage C-terminal to Lys and Arg) with a maximum of 2 missed cleavages permitted. Deamidation of Asn and Gln, oxidation of Met, pyro-Glu (with peptide N-term Gln), and protein N-terminal acetylation were set as variable modifications. Carbamidomethyl on Cys was searched as a fixed modification. We used the MaxLFQ algorithm for LFQ, integrated into the MaxQuant environment^56,58^. MaxQuant output was processed and statistical tests performed using the R software package (version 3.4.3). Heatmaps were plotted using Tableau (version 2018.3.2).

### Statistics

We analyzed rat liver samples collected after 12 weeks of treatment without or with DSF (100 and 200 mg/kg BW). To these data we applied a Wilcox robust test to allow for proteins whose distribution for the difference between treatment groups across animals was not normally distributed. Fold changes comparing liver protein abundance after DSF treatment vs. control were calculated using the median. The Pearson correlation was calculated between all physiological measures and all measured proteins, and the associated *p*-values were adjusted using the BH correction to control for multiple testing. For all data sets, statistical analyses were performed using R (version 3.4.3) and processed data were plotted using Tableau (version 10.0.2). Data are represented as box plots, unless otherwise stated. Significance was set at *P* < 0.05.

#### Protein extraction, gel electrophoresis, and western blotting

Mouse liver tissues were collected and snap frozen in liquid nitrogen. Liver was then homogenized in RIPA buffer (Boston Bioproducts) supplemented with protease and phosphatase inhibitor cocktails (Sigma-Aldrich) and 10 µM trichostatin A (Sigma-Aldrich) using the Kinetmatica Polytron Homogeneizer (Thermo Fisher Scientific) on ice. After 30 min sitting on ice, homogenates were centrifuged at 14,000 r.p.m. for 10 min at 4 °C. Protein determination was performed on the clarified lysates using the Bradford reagent (BioRad) and were then mixed with 4× laemmli sample buffer. Samples (10 μg per lane) were resolved on pre-casted gradient SDS-polyacrylamide gel electrophoresis gels (Biorad) under reducing conditions and electrotransferred to nitrocellulose membranes. Membranes were blocked for 1 h in PBS-T (10 mM phosphate-buffered saline/0.05% Tween-20) supplemented with 5% bovine serum albumin, then incubated overnight at 4 °C with the primary antibody of interest. The membranes where then incubated with secondary antibodies conjugated with horseradish peroxidase (1 : 5000 in PBS-T/5% non-fat milk) for 1 h at room temperature. Immunoreactive bands were visualized using ECL Plus Western Blotting Detection System and the bands were quantified by scanning densitometry (ImageJ software). Membranes were stained with Ponceau S prior to the blocking step and each band was normalized to the total densitometric value of the Ponceau staining for that line. Alternatively, each band was normalized to a loading control such as glyceraldehyde 3-phosphate dehydrogenase (GAPDH). All blots or gels were derived from the same series of experiments and were processed in parallel; *n* = 5 per group; age = 57 weeks; diet = 41 weeks.

The source of the antibodies used for immunoblotting were as followed: IGFBP2 (Novus, catalog number Nbp1-57914, 1 : 1000 dilution), phosphorylated p65Rel (Cell Signaling, catalog number 3039S, 1 : 1000 dilution), total p65 (Epitomics, catalog number 1546-1, 1 : 10,000 dilution), IL-1β (Cell Signaling, catalog number 12507, 1 : 1000 dilution), acetylated (Abcam, catalog number ab137037), and total SOD2 (Abcam, catalog number ab13533, 1 : 5000 dilution for both antibodies), SIRT1 (Sigma, catalog number S5196, 1 : 2500 dilution), SIRT3 (Cell Signaling, catalog number 5490, 1 : 1000 dilution), GAPDH (Santa Cruz, catalog number sc-32233, 1 : 5000 dilution), acetyl-lysine (EMD Millipore, catalog number 05-515, 1 : 1000 dilution), and 4-HNE (EMD Millipore, catalog number 393206, 1 : 1000 dilution).

#### Measurement of GST activity

The hepatic cytosolic fraction (free of microsomes) was prepared following the method described by Mach et al.^59^ and protein concentration was determined by Bradford method. The GST activity toward DCNB (1,2-dichloro-4-nitrobenzene) was measured according to an established method^60^. In brief, diluted cytosolic extract was mixed with glutathione and GST activity was measured over 5 min upon addition of the substrate DCNB to produce a detectable conjugate measured at 340 nm absorbance at room temperature.

#### Cell cultures

Mouse fibroblasts (NIH3T3) were obtained from the American Type Culture Collection (Manassas, VA). All cells were maintained in Dulbecco’s modified Eagle’s medium (Sigma-Aldrich, St-Louis, MO) in the presence of 10% newborn calf serum, 50 μg/ml penicillin, and 50 μg/ml streptomycin in a humidified incubator at 37 °C with 5% CO_2_.

#### Macroautophagy analysis

Similar to previous studies, macroautophagy activity was measured in intact cells upon transduction with lentivirus carrying the mCherry-GFP-LC3 tandem construct. Cells were plated on coverslips or glass-bottom 96-well plates and fluorescence was read in both red and green channels. Puncta positive for both fluorophores correspond to APGs, whereas those only positive for the red fluorophore correspond to AUTs. Autophagic flux was determined as the conversion of APGs (yellow) to AUTs (red only puncta)^41^.

#### High-content microscopy

Similar to previous studies, high-content microscopy was performed as reported by Hernandez et al.^41^. Cells plated in glass-bottom 96-well plates were treated for the indicated times and, after fixation with 4% paraformaldehyde, images were acquired using a high-content microscope (Operetta, Perkin-Elmer). Images of nine different fields per well were captured, resulting in an average of 2500–3000 cells per condition. Nuclei and puncta were identified using the manufacturer’s software. The number of particles/puncta per cell was quantified using the “particle identifier” function in the cytosolic region after thresholding in non-saturated images^39,61^. In all cases, focal plane thickness was set at 0.17 μm and sections with maximal nucleus diameter were selected for quantification. Values are presented as number of puncta per cell section. Under our acquisition conditions, these values represent ~10–20% of the total puncta per cell.

#### Intracellular protein degradation

The degradation of long-lived proteins was measured as reported by Hernandez et al.^41^. In brief, confluent cells were labeled with ^3^H-leucine (2 μCi/ml, Perkin-Elmer, Waltham, MA) for 48 h at 37 °C and then extensively washed and maintained in complete (10% fetal bovine serum) or serum-deprived medium containing an excess of unlabeled leucine (2.8 mM) to prevent reutilization of radiolabeled leucine^61,62^. Aliquots of the media taken at different times were precipitated with TCA and proteolysis was measured as the percentage of the initial acid-insoluble radioactivity (protein) transformed into acid-soluble radioactivity (amino acids and small peptides) at the end of the incubation. Total radioactivity incorporated into cellular proteins was determined as the amount of acid-precipitable radioactivity in labeled cells immediately after washing.

### Statistical analysis

No statistical method was used to predetermine sample size. All statistical analyses were done with two-tailed unpaired Student’s *t*-test or one-way ANOVA, followed by Tukey’s post hoc comparison, unless otherwise stated. These analyses reflect fold change from SD- or HFD-fed control animals and were performed using either Excel for Mac (Microsoft Corp.) or Prism v.6 (GraphPad Software). Data are expressed as means ± SEM. Values of *P* ≤ 0.05 were considered statistically significant.

### Reporting summary

Further information on research design is available in the Nature Research Reporting Summary linked to this article.

## Supplementary information

reporting summary

SI

## Data Availability

The data sets generated during and/or analyzed during the current study are available from the corresponding author, Rafael de Cabo (decabora@mail.nih.gov), on reasonable request. This study did not generate new unique reagents. Our microarray data have been deposited in the Gene Expression Omnibus database (https://www.ncbi.nlm.nih.gov/geo) with the following accession number GEO: GSE110200. RAW MS data from our liver proteome analysis have been deposited to the ProteomeXchange Consortium (http://proteomecentral.proteomexchange.org) via the PRIDE partner repository with the data set identifier PXD016793, username: reviewer58131@ebi.ac.uk, password: U2nPrkRn.
