# Supplementary material for: Elucidating the mechanisms by which disulfiram protects against obesity and metabolic syndrome
Source: NPJ Aging Mech Dis. 2020 Jul 21;6:8. doi: 10.1038/s41514-020-0046-6 (PMC7374720; doi:10.1038/s41514-020-0046-6)
Supplement: Supplementary file 2 — SI [file 41514_2020_46_MOESM2_ESM.pdf]

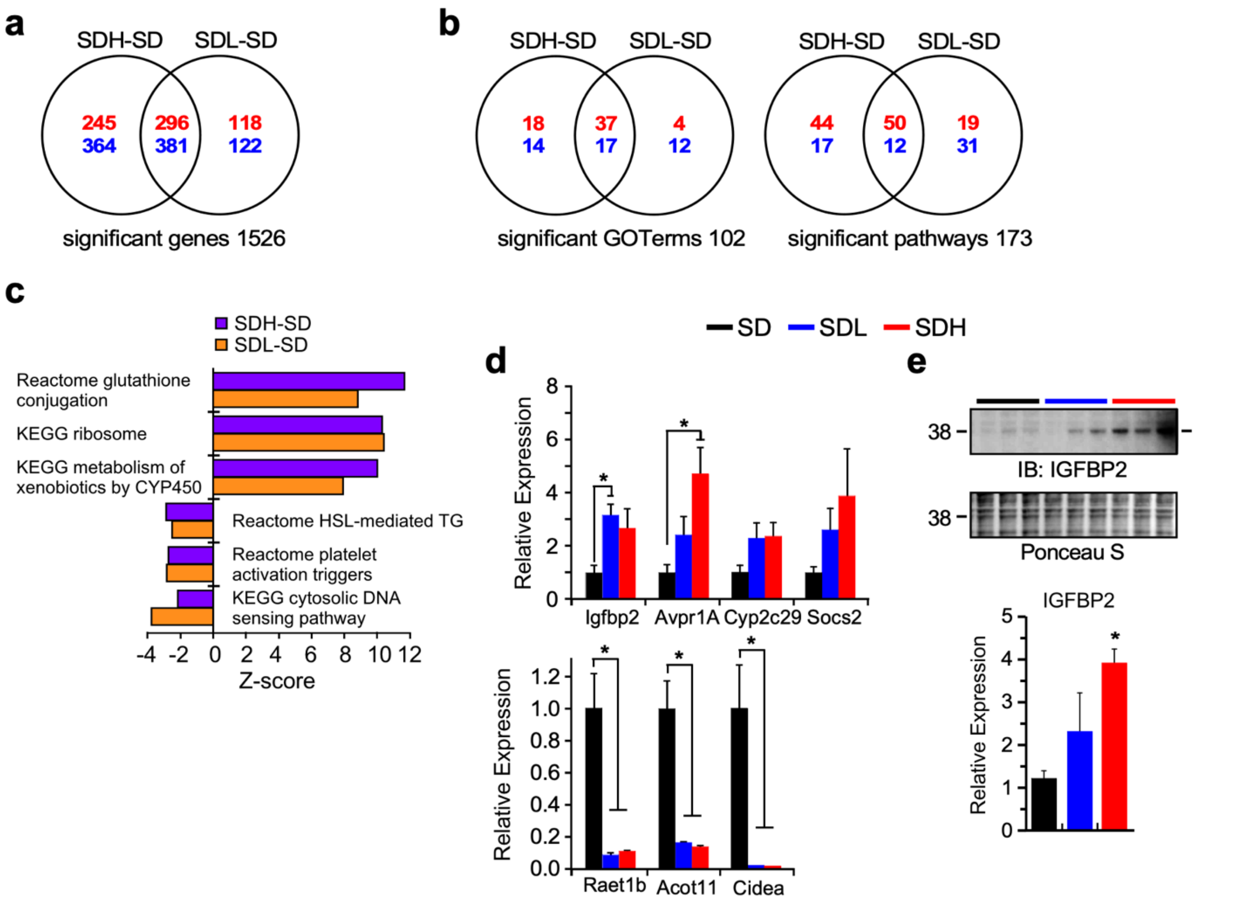

Supplementary Fig. 1. Molecular alterations in the liver of standard diet-fed mice supplemented with DSF. a Venn diagrams depicting genes significantly upregulated (red) and downregulated (green) in SD livers in response to low and high doses of DSF. **b** Venn diagrams depicting GO terms and canonical pathways significantly upregulated (red) or downregulated (green) in the SDH_SD and SDL_SD pairwise comparisons. **c** Enrichment of select pathways shared between the SDH_SD and SDL_SD pairwise comparisons. **d** Validation of microarray data by quantitative real-time PCR. *n* = 5 per group. Values were normalized to SD controls. **e** Liver extracts were prepared from mice after 40 weeks on the indicated dietary intervention and immunoblotted for IGFBP2 (upper panel). Relative IGFBP2 protein expression after data normalization using Ponceau S staining of the membrane is depicted in the lower panel. *n* =3 per group. *, $P \leq 0.05$. Related to Fig. 1

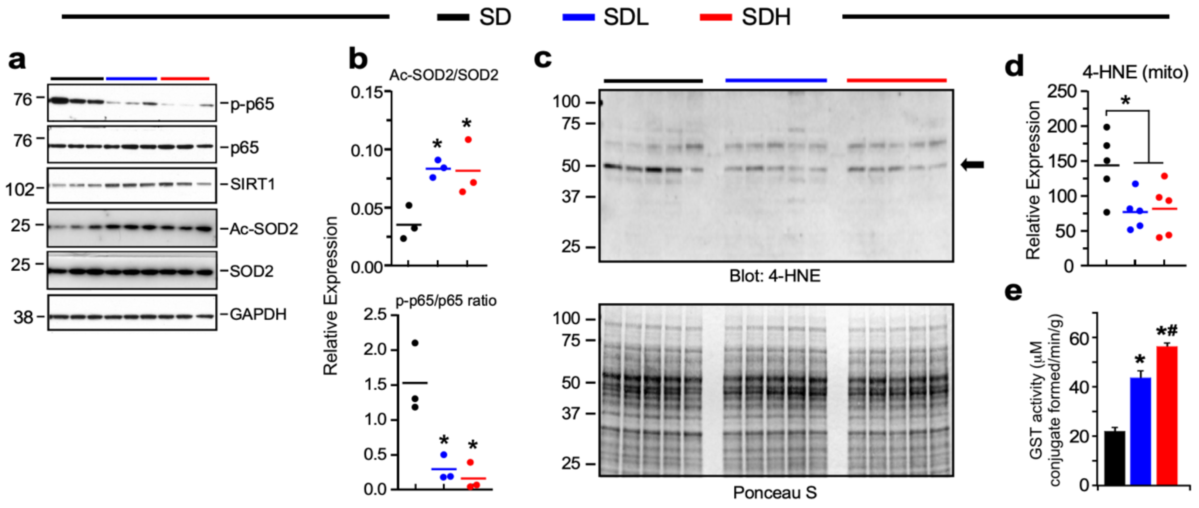

Supplementary Fig. 2. Molecular and biochemical alterations in the liver of SD-fed mice supplemented with DSF. a Immunoblotting of liver lysates for total and phosphorylated forms of p65Rel A, total and acetylated forms of SOD2, and SIRT1. **b** Relative acetylated/total ratio for p65Rel A and SOD2 after data normalization using GAPDH as loading control. **c** Detection of 4-HNE-conjugated proteins in liver homogenates. Ponceau S staining of the membrane confirmed equal protein load. Molecular mass markers (in kDa) are depicted on the left of immunoblots. **d** Densitometric analysis of 4-HNE signals shown in **c**. **e** GST activity measured in liver microsomal fractions. * $P \leq 0.05$ versus SD; #, $P \leq 0.05$ versus SD + low DSF. Related to Fig. 2.

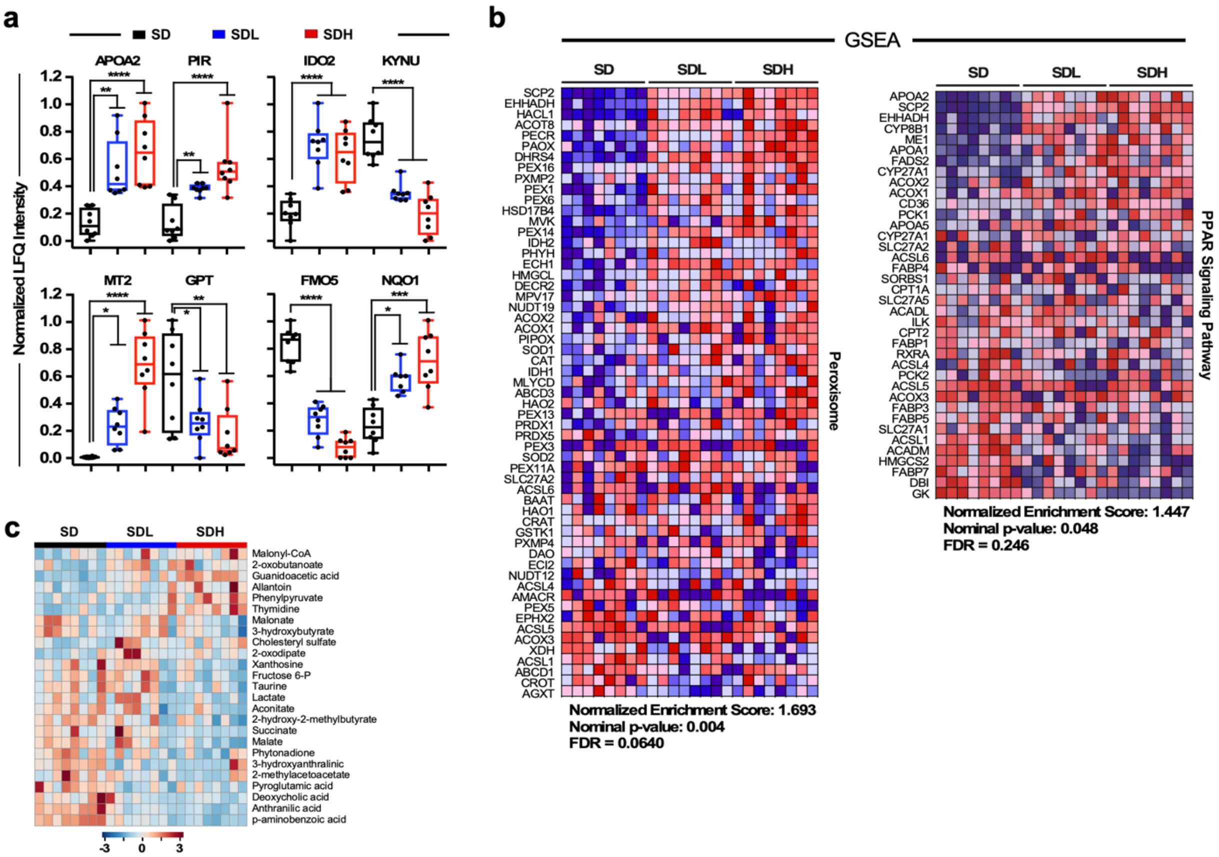

Supplementary Fig. 3. Treatment with DSF for 12 weeks alters liver proteome and serum metabolome in chow-fed rats. a Abundance of a select group of proteins significantly impacted by DSF treatment. Normalized LFQ intensity values are represented in box and whisker plot format (n = 8 per group). Statistics for the effects of DSF intervention represent the p-value from a one-way ANOVA with Dunnett's post-hoc tests. *, **, ***, ****, $P < 0.05$, 0.01, 0.001, and 0.0001. **b** Gene Set Enrichment Analysis (GSEA) depicting two sets of proteins whose expression was significantly impacted by DSF supplementation. These sets of proteins were grouped together based on their involvement in peroxisome (*left panel*) and PPAR signaling pathway (*right panel*). **c** Heatmap of the 25 metabolites in the rat serum metabolome that contributed to the separation between control and DSF-treated groups. Related to Fig. 3.

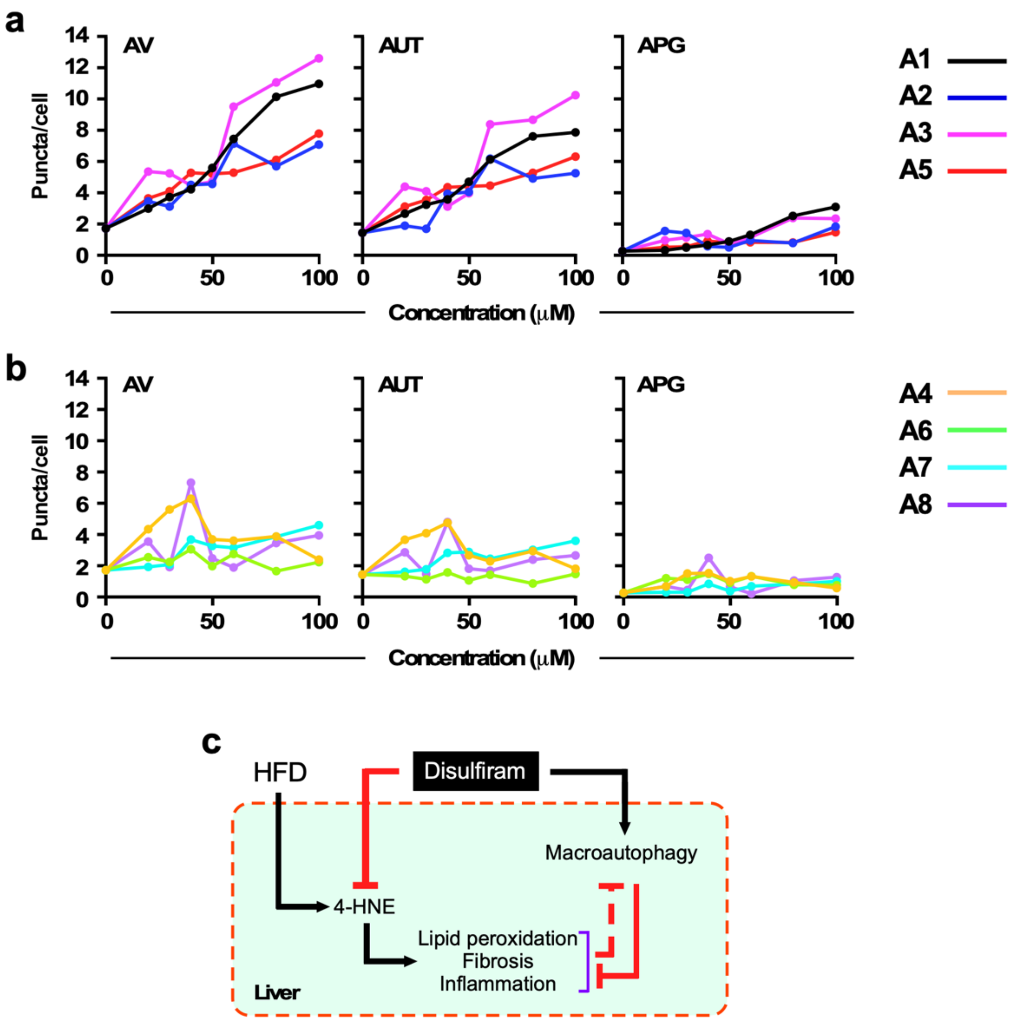

Supplementary Fig. 4. Structure-activity of DSF derivatives on autophagy. a,b NIH3T3 cells expressing the mCherry-GFP-LC3 reporter were exposed to the indicated concentrations of DSF (A1) and dithiocarbamate analogs (A2-A8) for 24 h and the changes in the number of autophagic vacuoles (AV), autolysosomes (AUT) or autophagosomes (APG) was quantified using high-content microscopy. Compounds with potent stimulatory effect on autophagy are shown in **a** and those with a more discrete effect in **b**. All values are mean of 4 individual wells and quantifications were done in at least 2500 cells per condition in three different experiments using high-content microscopy. Abbreviations: A1, bis(diethylthiocarbamate) disulfide aka disulfiram; A2, ammonium pyrrolidinedithiocarbamate; A3, Mn^{2+}-Zn^{2+} ethylenebis(dithiocarbamate); A4, Mn^{2+} ethylenebis(dithiocarbamate) aka pestanal; A5, Na^{+} diethyldithiocarbamate trihydrate; A6, triethylammonium *N*-(3,4-dichlorophenyl) dithiocarbamate; A7, *S*-cyanomethyl-*N*-methyl-*N*-(pyridin-4-yl) dithiocarbamate; A8, Zn^{2+} dimethyldithiocarbamate. **c** Schematic model of the proposed dual mechanism of action of DSF in its protective effect against high-fat diet (HFD). The cellular processes inhibited through the effect of DSF on 4-HNE levels and autophagy are indicated. Related to Fig. 4.

Supplementary Table 1. Partial list of genes reciprocally affected in response to DSF supplementation in male C57BL/6 mice fed either an HFD or SD diet. Related to Fig. 1.

		zratio		
Gene name	Definition/Function	HFD-SD	HFDH-HFD	HFDL-HFD
Cyp2b9	Oxidoreductase activity toward steroids, FA and xenobiotics	**17.65**	**-9.00**	*-7.82*
Upp2	Liver-specific uridine phosphorylase 2	**5.76**	**-4.01**	*-0.89*
Gstm2	GST-class Mu - detoxification of electrophilic compounds	**5.10**	**-2.57**	*-3.36*
Jak3	Cytokine receptor-mediated intracellular signal transduction	**3.27**	**-1.64**	*-0.85*
Cyp2b13	Oxidoreductase activity	**17.92**	**-16.03**	**-14.29**
Cidec	Binds to lipid droplets and regulates their enlargement	**6.47**	**-7.36**	**-9.56**
Hk2	Hexokinase 2 - involved in the increased rate of glycolysis	**6.18**	**-6.36**	**-7.33**
Tceal8	Transcription elongation factor A-like 8	**5.13**	**-7.18**	**-8.22**
Raetib	Retinoic acid early transcript beta – macrophage activation	**4.52**	**-12.08**	**-11.58**
Vldr	Very low-density lipoprotein receptor – triglyceride metab.	**3.93**	**-6.08**	**-6.50**
Spp1	Osteopontin – acts as a cytokine by reducing IL-10 production	**3.60**	**-3.85**	**-6.90**
Hsd3b5	3β-HSD - Biosynthesis of hormonal steroids	**-20.04**	**15.81**	**20.75**
Igfbp2	IGF binding protein 2 – prolongs IGFs' half-life	**-4.27**	**8.13**	**9.14**
Cyp7b1	Homeostasis of cholesterol, bile acids and oxysterols	**-3.99**	**5.85**	**6.27**
Cdh1	E-cadherin – mechanisms regulating cell-cell adhesion	**-3.58**	**5.70**	**4.43**
Elovl3	Elongation of very long chain fatty acids, e.g. C18-acylCoA	**-6.58**	**2.43**	*-0.12*
C6	Key role in the innate and adaptive immune response	**-4.73**	**5.75**	*4.37*
Mup21	Major urinary protein 21 – pheromone ligand	**-3.98**	**4.75**	*3.91*
Gstp1	GST-class Pi – conjugation of reduced glutathione to electrophiles.	**-3.61**	**6.06**	*3.59*

Gene name	Definition/Function	SDH-SD	SDL-SD
Cyp2b20	aka Cyp2b6. Steroid, ketone and xenobiotic metabolism	**18.41**	**24.34**
Tff3	trefoil factor 3 promotes the mobility of epithelial cells in healing proc.	**12.99**	**12.41**
Mt2	Melatonin Receptor 1B	**12.41**	**9.23**
Lpl	lipoprotein lipase	**11.04**	**6.09**
Cxcl1	Chemokine (C-X-C Motif) Ligand 1	**9.37**	**9.09**

Hsd17b9	retinol dehydrogenase 5	**9.37**	**9.71**
Gsta2	glutathione S-transferase alpha 2	**9.04**	**5.50**
Slc39a4	solute carrier family 39 (zinc transporter). Zinc uptake	**8.60**	**12.54**
Rbp1	retinol binding protein 1, cellular	**8.51**	**6.97**
Socs3	suppressor of cytokine signaling 3. JAK2 inhibitor	**8.48**	**7.14**
Cyp2c29	Has aldehyde oxygenase activity. Xenobiotic metabolism	**7.96**	**9.76**
Cfd	complement factor D (adipsin), serine protease	**-15.23**	**-17.91**
Cidea	cell death-inducing DFFA-like effector a	**-14.49**	**-19.49**
Per2	period circadian clock 2	**-9.12**	**-5.72**
Raet1b	retinoic acid early transcript beta	**-8.29**	**-12.69**
Apoa4	apolipoprotein A-IV has a role in chylomicrons and VLDL	**-8.25**	**-9.43**
Ntrk2	catabolism	**-7.65**	**-7.41**
Clstn3	neurotrophic tyrosine kinase, receptor, type 2	**-7.24**	**-7.50**
Tgfb1i4	calsyntenin 3 stabilizes APP metabolism	**-6.85**	**-7.94**
	TGFβ-1-induced transcript 4 acts as a transcriptional repressor		

HFD, high-fat diet; HFDH, HFD+high DSF; HFDL, HFD+low DSF. SD, standard diet; SDH, SD+high DSF; SDL, SD+low DSF. **Boldface**: significant expression; *italic*, non-significant.

Supplementary Table 2. Partial list of GOTerms and pathways significantly affected in response to DSF supplementation in male C57BL/6 mice fed either a HFD or SD diet. Related to Fig. 1.

	Zscore		
GO Term	HFD-SD	HFDH-HFD	HFDL-HFD
02504-Antigen processing and presentation of peptide or polysaccharide antigen via MHC class II	**4.737**	**-4.336**	0
19886-Antigen processing and presentation of exogenous peptide antigen via MHC class II	**4.582**	**-3.971**	0
42613-MHC Class II protein complex	**4.352**	**-3.973**	0
05764-Lysosome	**4.037**	**-3.471**	**-3.591**
08152-Metabolic process	**3.131**	**-6.379**	**-5.577**
04029-Aldehyde dehydrogenase (NAD) activity	**2.896**	**-5.226**	0
06631-Fatty acid metabolic process	**2.774**	**-4.688**	**-2.794**
05634-Nucleus	**-5.452**	**2.042**	**2.494**
03676-Nucleic acid binding	**-5.097**	**1.991**	**3.556**
06355-Regulation of transcription DNA-dependent	**-4.066**	**2.662**	**3.336**
06350-Transcription	**-3.927**	**1.892**	**2.881**
45449-Regulation of transcription	**-3.218**	**3.073**	**1.785**
05667-Transcription factor complex	**-3.077**	**2.576**	**2.230**
Pathway	HFD-SD	HFDH-HFD	HFDL-HFD
Reactome metabolism of amino acids	**4.736**	**-2.213**	0
KEGG valine leucine and isoleucine degradation	**4.581**	**-4.658**	**-3.876**
Reactome regulation of insulin secretion	**3.950**	0	**-4.021**
KEGG oxidative phosphorylation	**3.487**	0	**-1.994**
Reactome electron transport chain	**3.377**	0	**-3.005**
KEGG propanoate metabolism	**3.167**	**-5.246**	**-5.352**
KEGG peroxisome	**2.849**	**-3.338**	**-2.287**
KEGG butanoate metabolism	**2.555**	**-4.475**	**-4.517**
Reactome mitochondrial FFA β-oxidation	**2.506**	**-2.631**	0
KEGG lysosome	**2.368**	0	**-3.134**

GO Term		SDH-SD	SDL-SD
04364-Glutathione transferase activity		**9.188**	**7.311**
05830-Cytosolic ribosome		**7.805**	**7.711**
06953-Acute phase response		**7.334**	**7.250**
16712-Oxidoreductase activity acting on paired donors		**7.202**	0
50381-Unspecific monooxygenase activity		**6.305**	0
06695-Cholesterol biosynthetic process		**4.970**	0
02504-Antigen processing and presentation of peptide or polysaccharide antigen via MHC class II		**-3.320**	0
42613-MHC Class II protein complex		**-3.232**	0

32259-Methylation		**-2.768**	0
06886-Intracellular protein transport		**-3.565**	**-2.463**
05739-Mitochondrion		**-3.564**	**-6.141**
09374-Biotin binding		**-3.274**	**-3.017**
16573-Histone acetylation		**-2.169**	**-2.411**
Pathway		SDH-SD	SDL-SD
Reactome Glutathione conjugation		**11.700**	**8.850**
Reactome peptide chain elongation		**10.530**	**10.617**
KEGG ribosome		**10.365**	**10.457**
KEGG metabolism of xenobiotics by cytochrome p450		**10.060**	**7.968**
Reactome cholesterol biosynthesis		**8.515**	0
KEGG steroid biosynthesis		**5.490**	0
KEGG Snare interactions in vesicular transport		**-2.092**	0
Reactome hormone-sensitive lipase HSL-mediated TG		**-2.865**	**-2.552**
Reactome platelet activation triggers		**-2.767**	**-2.827**
KEGG cytosolic DNA sensing pathway		**-2.163**	**-3.761**

HFD, high-fat diet; HFDH, HFD+high DSF; HFDL, HFD+low DSF. SD, standard diet; SDH, SD+high DSF; SDL, SD+low DSF. **Boldface**: significant expression; The entry '0' indicates no significant difference.

Supplementary Table 3. Partial list of genes whose expression levels in response to DSF supplementation were shared in male C57BL/6 mice fed both SD and HFD diets. Related to Fig. 1.

	Zratio				
Gene name	HFD-SD	HFDH-HFD	HFDL-HFD	SDH-SD	SDL-SD
Raet1b	**4.52**	**-12.08**	**-11.58**	**-8.29**	**-12.69**
Raet1b	**4.00**	**-10.52**	**-10.84**	**-6.93**	**-10.66**
Lgals1	**3.16**	**-10.33**	**-11.17**	**-4.85**	**-5.80**
1600023A02R	**3.06**	**-10.44**	**-9.45**	**-8.55**	**-5.26**
LOC268782	**1.51**	**-2.89**	**-2.60**	**-2.01**	**-3.03**
Igfbp2	**-4.27**	**8.13**	**9.14**	**5.39**	**6.46**
Cdh1	**-3.58**	**5.70**	**4.43**	**4.88**	**4.89**
Avpr1a	**-3.20**	**6.14**	**5.89**	**4.04**	**3.61**
Avpr1a	**-3.04**	**9.33**	**8.35**	**6.30**	**5.08**
Csad	**-2.96**	**3.85**	**3.78**	**5.87**	**7.23**
Abca8a	**-2.77**	**3.60**	**3.11**	**2.22**	**2.78**
Cyp2b20	*6.15*	**15.99**	**17.87**	**18.41**	**24.34**
Igfbp2	*-3.20*	**8.80**	**10.16**	**6.12**	**6.83**
Socs2	*3.24*	**8.32**	**5.38**	**7.46**	**7.72**
Slco2a1	*-1.36*	**5.73**	**5.45**	**6.42**	**7.79**
Cyp2c29	*2.01*	**4.95**	**4.73**	**7.96**	**9.76**
Cyp1a2	*-0.59*	**4.92**	**5.83**	**2.85**	**3.62**
3110082I17Rik	*1.31*	**4.71**	**3.79**	**4.41**	**3.92**
Cyp1a2	*-0.50*	**4.61**	**5.76**	**2.25**	**2.62**
Apom	*0.01*	**4.43**	**3.40**	**4.16**	**3.92**
Ptpre	*0.61*	**4.03**	**3.15**	**2.61**	**2.09**
Cyp2c37	*1.45*	**4.01**	**3.65**	**2.90**	**2.36**
Iap	*-0.83*	**3.93**	**3.60**	**2.75**	**1.91**
Pnp	*0.61*	**3.85**	**2.07**	**2.47**	**2.06**
Foxq1	*1.02*	**3.50**	**2.91**	**4.01**	**6.22**
H13	*-0.68*	**3.43**	**1.74**	**1.57**	**1.73**
Rpl12	*0.22*	**3.42**	**2.36**	**3.33**	**3.41**
Cyp4f13	*-0.20*	**3.34**	**2.68**	**1.74**	**2.24**
Zfp259	*-0.08*	**3.26**	**2.73**	**2.95**	**1.96**
Cyp2c50	*1.46*	**3.17**	**3.44**	**2.96**	**2.42**
Rps10	*-0.03*	**3.11**	**1.68**	**1.62**	**1.68**
Cidea	*1.64*	**-16.66**	**-19.16**	**-14.49**	**-19.49**
Cfd	*-0.79*	**-12.72**	**-15.73**	**-15.23**	**-17.91**
Mmd2	*3.94*	**-12.28**	**-12.47**	**-6.24**	**-6.88**
Clstn3	*-0.25*	**-10.35**	**-10.57**	**-7.24**	**-7.50**
Robo1	*2.53*	**-8.49**	**-8.50**	**-2.86**	**-4.58**
Ntrk2	*-1.65*	**-8.06**	**-9.01**	**-7.65**	**-7.41**
Bhmt	*-0.17*	**-7.58**	**-6.42**	**-2.90**	**-5.23**

Slc17a8	*-2.38*	**-7.57**	**-3.09**	**-4.22**	**-4.25**
Anxa2	*3.07*	**-6.86**	**-8.69**	**-3.88**	**-5.15**
Lyplal1	*0.18*	**-5.55**	**-4.47**	**-1.98**	**-2.71**
Acot11	*-1.52*	**-5.45**	**-4.01**	**-5.87**	**-5.79**
1110028A07R	*1.38*	**-4.99**	**-5.10**	**-3.05**	**-2.42**
Rgs5	*0.96*	**-4.56**	**-3.25**	**-2.06**	**-1.94**
S100a11	*1.90*	**-4.19**	**-4.87**	**-2.34**	**-3.30**
Cd59b	*-0.29*	**-4.16**	**-3.98**	**-2.62**	**-2.12**
BC026585	*1.26*	**-4.07**	**-5.32**	**-2.13**	**-2.80**
Slc44a1	*-0.12*	**-3.98**	**-3.33**	**-1.81**	**-2.04**
1300018L09Rik	*-1.39*	**-3.95**	**-1.83**	**-1.59**	**-1.61**
1300013J15Rik	*0.39*	**-3.94**	**-2.54**	**-5.08**	**-6.14**
1300013J15Rik	*0.96*	**-3.90**	**-2.90**	**-5.45**	**-5.74**

HFD, high-fat diet; HFDH, HFD+high DSF; HFDL, HFD+low DSF; SD, standard diet; SDH, SD+high DSF; SDL, SD+low DSF. **Boldface**, significant expression; *italic*, non-significant.

Supplementary Table 4. List of the top 20 significantly changed proteins in the livers of rat treated with low (L-DSF) and high (H-DSF) doses of DSF vs. control chow (ctrl). FC, fold change. Related to Fig. 3.

				Log10P_BH		Log2FC	
**Protein.IDs**	**Protein.names**	**Gene.names**	**Global_Log10P_BH**	**H-DSF-crtl**	**L-DSF-crtl**	**H-DSF-crtl**	**L-DSF-crtl**
P46418;A0A0G2JTB1;A0A0G2K8Q5	Glutathione S-transferase alpha-5	Gsta5	5.91	6.58	3.22	**4.00**	**3.33**
B2GV28;A0A140UHW7;P00176;F1LMN1;	Cytochrome P450 2B1	Cyp2b1	2.64	3.16	1.53	**2.91**	**2.63**
P38918	Aflatoxin B1 aldehyde reductase member 3	Akr7a3	3.49	4.03	2.06	**2.45**	**1.70**
Q6T5E9;Q63662		Ugt1a1;Ugt1a6	4.08	4.53	2.68	**2.29**	**1.86**
P09875;A0A0G2JUD3	UDP-glucuronosyltransferase 2B1	Ugt2b1	4.11	4.68	2.40	**2.07**	**1.75**
Q6T5E8;Q6T5E7		Ugt1a1	2.72	3.19	1.62	**1.88**	**1.54**
Q811X6	Lambda-crystallin homolog	Cryl1	2.43	2.89	1.56	**1.85**	**1.50**
P04903;Q4FZZ3	Glutathione S-transferase alpha-2;Glutathione S-transferas	Gsta2;Gsta5	6.11	6.68	3.19	**1.58**	**1.03**
Q5M827	Pirin	Pir	3.03	3.59	1.63	**1.38**	**1.11**
Q32Q55	Carboxylic ester hydrolase	Ces2h	6.92	7.29	5.19	**1.30**	**0.96**
A0A0H2UI26;Q5RKL5	Metalloreductase STEAP3	Steap3	5.26	5.31	4.42	**-1.07**	**-0.95**
F1LQQ8;P06760;Q7TPJ3	Beta-glucuronidase	Gusb	9.62	9.45	8.98	**-1.13**	**-1.04**
Q8K4C0	Dimethylaniline monooxygenase [N-oxide-forming] 5	Fmo5	7.92	8.38	5.99	**-1.14**	**-0.72**
Q5I0N0;Q9QZH8	Arylacetamide deacetylase	Aadac	4.35	4.91	2.67	**-1.18**	**-0.60**
Q60587;A0A0G2K330	Trifunctional enzyme subunit beta, mitochondrial;3-ketoacy	Hadhb	8.76	9.16	6.95	**-1.19**	**-0.83**
Q64428	Trifunctional enzyme subunit alpha, mitochondrial;Long-cha	Hadha	9.12	9.45	7.37	**-1.22**	**-0.86**
M0RB46;Q5I0E2;F1LQ59	PHD finger protein 11	Phf11b;Phf11	2.56	2.46	2.33	**-1.28**	**-1.68**
Q5FVR5;A0A0G2JV92;A0A0G2K2H6	Acyl-coenzyme A amino acid N-acyltransferase 2	Acnat2	3.86	4.15	2.83	**-1.38**	**-0.97**
P05183	Cytochrome P450 3A2	Cyp3a2	4.68	4.81	4.06	**-1.50**	**-1.34**
P17764	Acetyl-CoA acetyltransferase, mitochondrial	Acat1	8.47	8.60	7.08	**-1.75**	**-1.31**

Supplementary Table 5. Enrichment of KEGG pathways (from STRING) and GSEA report establishing a number of KEGG pathways statistically significant in livers of DSF-treated rats vs. controls. Related to Fig. 3.

# term ID	Term description	# Genes	Fdr
mmu01100	Matabolic pathways	31	6.94E-17
mmu00983	Drug metabolism – other enzymes	12	2.51E-14
mmu00980	Metabolism of xenobiotics by cytochrome P450	10	2.12E-12
mmu05204	Chemical carcinogenesis	10	3.80E-11
mmu00982	Drug metabolism – cytochrome P450	9	7.27E-11
mmu00380	Tryptophan metabolism	6	2.40E-07
mmu00071	Fatty acid degradation	6	3.73E-07
mmu01212	Fatty acid metabolism	6	3.73E-07
mmu00280	Valine, leucine and isoleucine degradation	6	5.04E-07
mmu00480	Glutathione metabolism	6	8.11E-07

KEGG pathway	# Genes	ES	NES	NOM *P*-val	Q-value
Drug_Metabolism_CYP450	35	0.747	1.689	0.000	0.055
Peroxisome	57	0.600	1.693	0.004	0.064
Snare_Interactions_in_Vesicular_Transport	19	0.571	1.696	0.004	0.077
Metabolism_of_Xenobiotics_by_CYP450	30	0.824	1.715	0.000	0.084
Retinol_Metabolism	16	0.783	1.617	0.006	0.094
Proteasome	37	0.642	1.785	0.018	0.117
Glutathione_Metabolism	34	0.686	1.718	0.000	0.124
PPAR_Signaling_Pathway	38	0.497	1.447	0.048	0.246
Tyrosine_Metabolism	17	0.586	1.463	0.082	0.249

Gene, number of genes in Geneset; ES, enrichment score; NES, normalized ES; NOM *P*-val, normalized *P* value; Q-value, false discovery rate with cut-off value of 0.25.

Supplementary Table 6. List of murine oligonucleotide primers used for validation of microarray analysis. Related to Fig. 1.

Target mRNA	Primer	Sequence (5' → 3')
Igfbp2	For<br>Rev	CAGACGCTACGCTGCTATCC<br>CCCTCAGAGTGGTCGTCATCA
Avpr1A	For<br>Rev	GCTGGCGGTGATTTTCGTG<br>GCAAACACCTGCAAGTGCT
Cyp2c29	For<br>Rev	CATCGACCTCCTCCCCACTAGC<br>GGTTGGGAAACTCCTTGCTGTCA
Socs2	For<br>Rev	AGTTCGCATTCAGACTACCTACT<br>TGGTACTCAATCCGCAGGTTAG
Raet1b	For<br>Rev	TGGACACTCACAAGACCAATG<br>CCCAGGTGGCACTAGGAG
Acot11	For<br>Rev	TGGGGAGCTGAGCATTGGA<br>GGCCGACACTAATGGTATGGT
Cidea	For<br>Rev	ATCACAACTGGCCTGGTTACG<br>TACTACCCGGTGTCCATTTCT
Hprt	For<br>Rev	TGGGAGGCCATCACATTGT<br>GCTTTTCCAGTTTCACTAATGACA
18S	For<br>Rev	GTAACCCGTTGAACCCCATT<br>CCATCCAATCGGTAGTAGCG

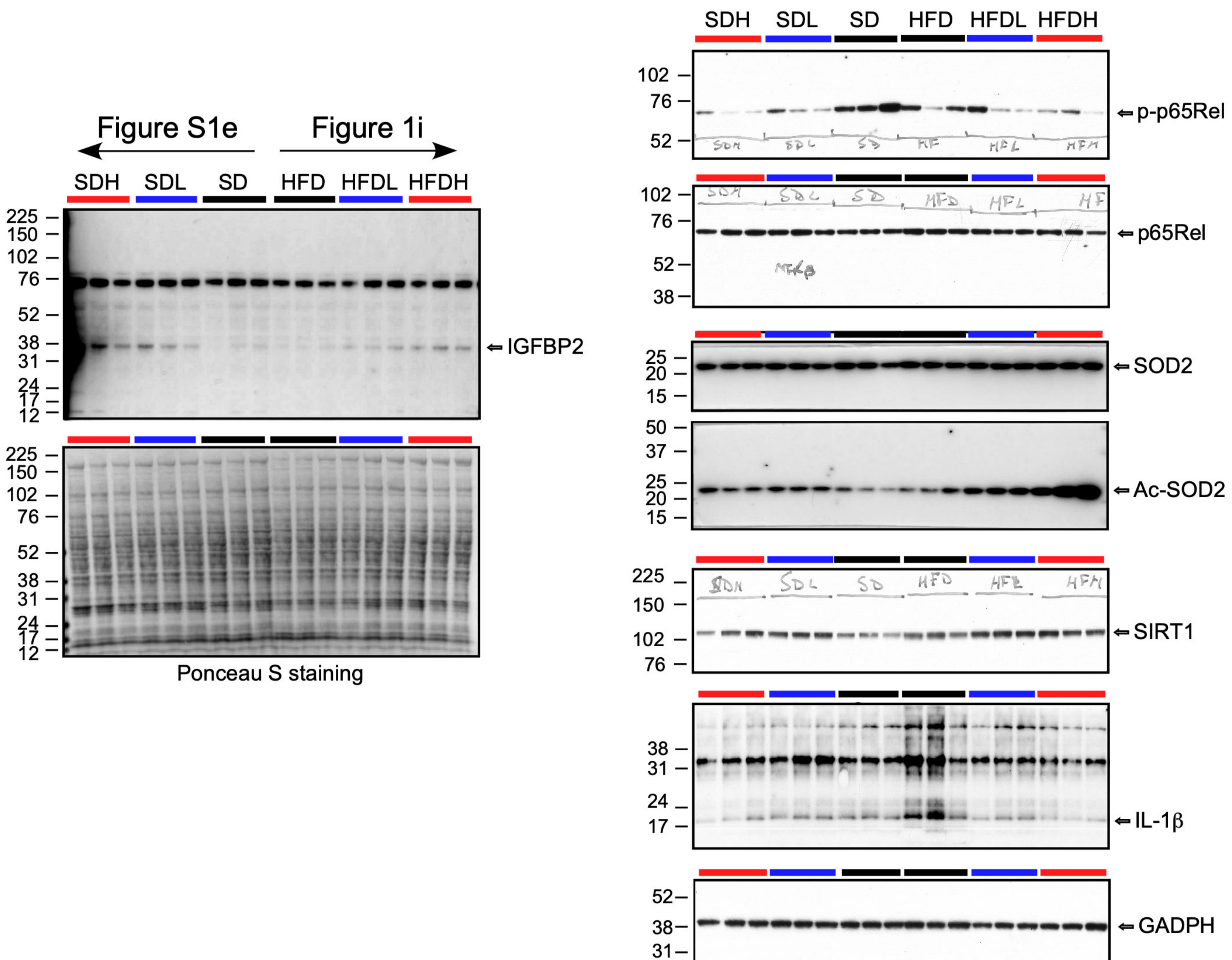
Figure S1e
Figure 1i
SDH
SDL
SD
HFD
HFDL
HFDH
225
150
102
76
52
38
31
24
17
12
IGFBP2
Ponceau S staining
p-p65Rel
p65Rel
50
37
25
20
15
SOD2
Ac-SOD2
SIRT1
IL-1β
GADPH
